# Re‐Purposing a Modular Origami Manipulator Into an Adaptive Physical Computer for Machine Learning and Robotic Perception

**DOI:** 10.1002/advs.202509389

**Published:** 2025-09-14

**Authors:** Jun Wang, Suyi Li

**Affiliations:** ^1^ Department of Mechanical Engineering Virginia Tech Blacksburg VA 24060 USA

**Keywords:** mechanical intelligence, perception, physical computing, soft robots

## Abstract

Physical computing has emerged as a powerful tool for performing intelligent tasks directly in the mechanical domain of functional materials and robots, reducing our reliance on the more traditional CMOS computers. However, no systematic study explains how mechanical design can influence physical computing performance. This study sheds insights into this question by repurposing an origami‐inspired modular robotic manipulator into an adaptive physical reservoir and systematically evaluating its computing capacity with different physical configurations, input setups, and computing tasks. By challenging this adaptive reservoir computer to complete the classical NARMA benchmark tasks, this study shows that its time series emulation performance directly correlates with the Peak Similarity Index (PSI), which quantifies the frequency spectrum correlation between the target output and reservoir dynamics. The adaptive reservoir also demonstrates perception capabilities, accurately extracting its payload weight and orientation information from the intrinsic dynamics. Importantly, such information extraction capability can be measured by the spatial correlation between nodal dynamics within the reservoir body. Finally, by integrating shape memory alloy (SMA) actuation, this study demonstrates how to exploit such computing power embodied in the physical body for practical, robotic operations. This study provides a strategic framework for harvesting computing power from soft robots and functional materials, demonstrating how design parameters and input selection can be configured based on computing task requirements. Extending this framework to bio‐inspired adaptive materials, prosthetics, and self‐adaptive soft robotic systems can enable next‐generation embodied intelligence, where the physical structure can compute and interact with its digital counterparts.

## Graphical abstract

Reservoir computing in the physical domain promises an alternative, more energy‐efficient approach to machine learning. However, the correlation between mechanical design and computing power remains elusive. This study sheds light on this gap by repurposing a meta‐stable modular origami manipulator into an adaptive computing kernel that can perform machine learning tasks typically handled by digital systems. Crucially, its switchable nature helps to uncover the connection between physical configuration and computing capacity. Extensive testing shows that spectral and spatial correlations among the body dynamics and training targets are key indicators for configuring a physical computer. These findings open promising avenues for reconfigurable robots and structures capable of physically and computationally adapting to their environment and task requirements.

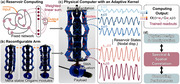

## 1 Introduction

Over the past few years, we have witnessed the rapid emergence of embodied physical computing.^[^ ^1^, ^2^ ^]^ The idea is to offload or “outsource” computation tasks from the electronic to the physical domain, significantly increasing the overall energy efficiency,^[^ ^3^ ^]^ parallelization,^[^ ^4^ ^]^ and resiliency against adversarial working conditions.^[^ ^5^ ^]^ To realize physical computing, researchers have explored two main strategies. One borrows the concept of algorithmic computing and uses bistable mechanisms as binary bits to construct logic gates for mathematical operations^[^ ^6^, ^7^ ^]^ (e.g., using mechanical AND, OR, and NOR gates to construct adder circuits^[^ ^8^ ^]^). Another strategy borrows the analog computing concept and uses dynamic and continuously variable signals to perform computation (e.g., using acoustic waves to solve differential equations^[^ ^9^ ^]^ or using mechanical vibrations to perform machine learning tasks^[^ ^10^, ^11^ ^]^). One can purposefully build such “algorithmic” or “analog” physical computers from the ground up, using meta‐material^[^ ^12^ ^]^ meta‐surface,^[^ ^13^ ^]^ or mechanical neural network architectures.^[^ ^14^ ^]^ On the other hand, one can also re‐purpose currently available physical systems—such as soft robots^[^ ^15^, ^16^ ^]^ and biological tissues^[^ ^17^, ^18^ ^]^—into computing devices, exploiting the computing capacity hidden behind their body dynamics. Implementing physical computation presents exciting potential to advance robotics, functional materials, wearable devices, smart infrastructures, and many other applications.^[^ ^19^ ^]^

**Figure 1:**
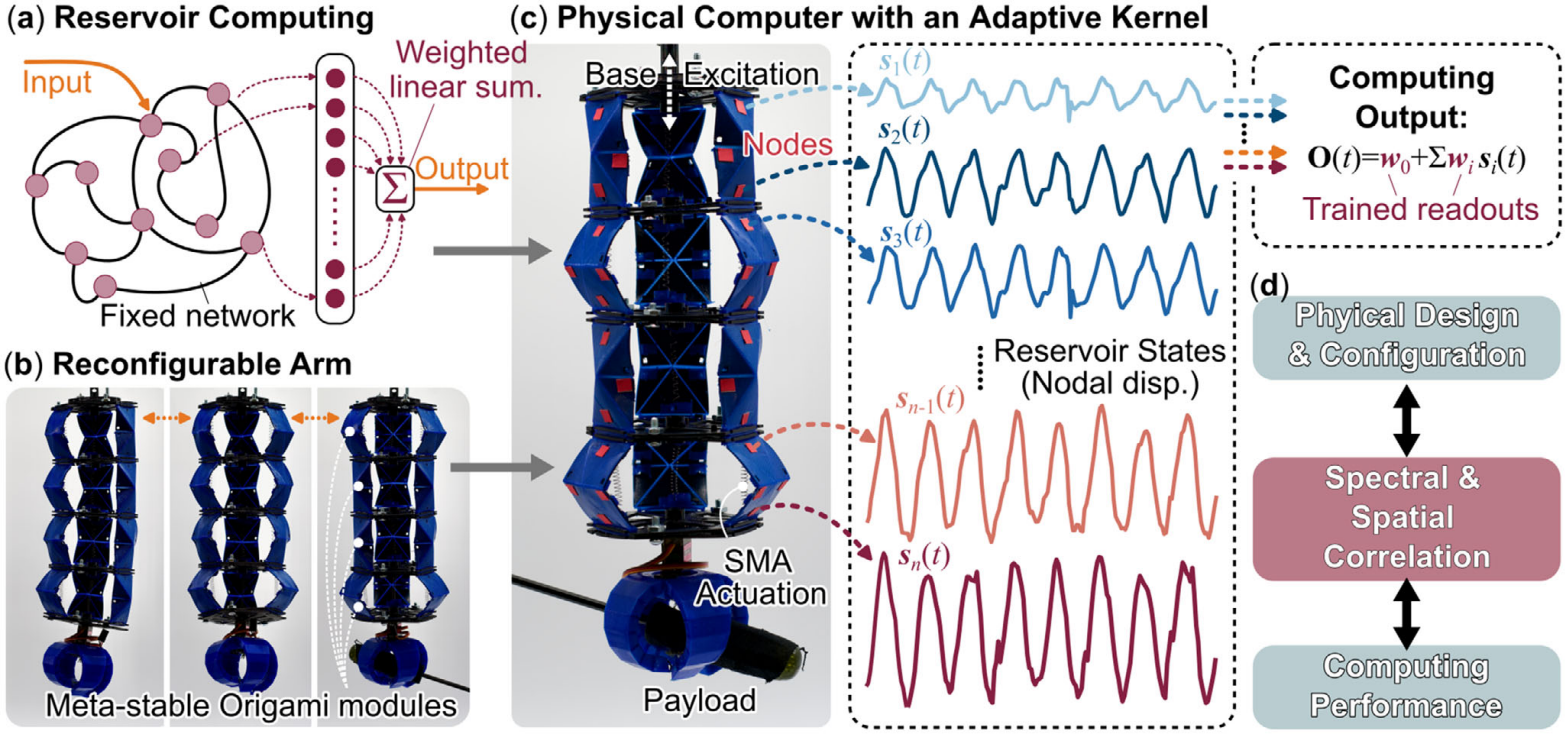
The overview of this study. a) A reservoir computer features a fixed neural network and a simple output layer of weighted linear summation. b) In this study, we employ a reconfigurable robotic arm consisting of origami‐inspired, meta‐stable modules as the physical platform. c) By applying the reservoir computing framework to this robotic arm, we create a physical computer with an adaptive kernel, which can be dynamically excited with base excitation or embedded shape memory alloy (SMA) coil actuators. The dynamics of this adaptive kernel are represented by the displacements of the markers attached throughout its body, which function as the reservoir state vectors *s*_*i*_(*t*). The reservoir's computing output is simply a weighted linear summation of these nodal displacements **O**(*t*) = *w*_0_ + ∑*w*_*i*_ *s*_*i*_(*t*), where the constant readout weights *w*_*i*_ will be trained according to the tasks at hand. d) In the big picture, this study reveals that the **spectral and spatial correlations** between the reservoir states and the targeted outcome are an informative hidden layer between the mechanical configuration of a physical computer and its computing performance.

Among these physical computing paradigms, the analog **P**hysical **R**eservoir **C**omputing (PRC) stands out because of its low power consumption, broad applicability, and impressive computing capability.^[^ ^20^ ^]^ The physical reservoir computing framework utilizes a physical system's dynamic responses to perform machine learning tasks.^[^ ^21^, ^22^ ^]^ This approach is a subset of reservoir computing, which itself is a special type of recurrent neural network (RNN) characterized by its fixed computing kernel. The core concept of physical reservoir computing is that a physical system capable of exhibiting a large degree of freedom and nonlinear dynamic responses can project an input excitation into a high‐dimensional state space (i.e., reservoir states). These state‐space vectors can then be “read out” to calculate the output through an analog and power‐efficient weighted linear summation (**Figure** **1**). Consequently, only the readout weights in the output layer need to be trained to achieve the desired input–output mapping for the machine‐learning task at hand. In essence, physical reservoir computing treats the physical system as a fixed neural network, with training occurring solely in the output layer. Using this reservoir computing framework, various physical systems have successfully been transformed into physical computers across multiple domains, including mechanical,^[^ ^23^ ^]^ electrical,^[^ ^24^, ^25^ ^]^ magnetic,^[^ ^26^ ^]^ photonic,^[^ ^27^ ^]^ and biological^[^ ^18^ ^]^ systems. These physical computers can emulate complex nonlinear time series,^[^ ^28^ ^]^ process sensory information to extract valuable knowledge (e.g., image recognition),^[^ ^29^, ^30^ ^]^ and generate control commands for robotic tasks.^[^ ^31^ ^]^

However, despite the promising potential of physical reservoir computing, it is still an emerging topic with many big‐picture questions to answer: What is the correlation between physical design and computing performance? How do we extract the most computing power from a physical structure? How can we accomplish more practical functions (beyond nonlinear time series emulation and simple sensory information perception)? Previous studies often apply physical systems “as is” for reservoir computing, without a deep understanding of the connection between computing and physical design. As a result, optimizing the physical setup for better computing performance becomes an ad‐hoc process, making it hard to understand the full extent of available computing power.

To shed insight into answering the aforementioned big‐picture questions, we apply the reservoir computing framework to an origami‐inspired, modular, and reconfigurable soft robotic manipulator, thus re‐purposing it into a physically *adaptive* reservoir kernel (Figure 1a–c).

Modular design is popular in robotics because of its scalability, reconfigurability, and versatility.^[^ ^32^ ^]^ In particular, we make each module multi‐stable using 3D‐printed and pre‐stressed origami panels so that each stable equilibrium has a unique shape and stiffness. Therefore, by adjusting the number of robotic modules and switching between their stable states, we can quickly adapt the physical reservoir's dynamic characteristics. Critically, the compliant origami structure naturally possesses nonlinearity, high‐dimensionally, and fading memory properties that are prerequisites of physical reservoir computing.^[^ ^33^ ^]^ Furthermore, folding‐induced nonlinearity in origami structures has been proven to be a reliable source of broadband and diverse nonlinear responses.^[^ ^34^, ^35^ ^]^ So origami structure is a capable physical computer that can perform complex time‐series emulation and information perception tasks in parallel.^[^ ^36^ ^]^ Therefore, a modular, origami‐based structure is an ideal platform for uncovering the relationship between physical configurations and computing performance.

Via a thorough analysis of the adaptive kernel's dynamics at different configurations, we discover two metrics that can serve as the linkages between physical setups (i.e., mechanical design and constitutive properties) and computation performance (Figure 1d). These metrics are (1) **spectral correlation** between the reservoir dynamics and targeted output and (2) **spatial correlation** within the reservoir's state space. These two metrics are frequently used in nonlinear structural dynamics analysis,^[^ ^37^, ^38^ ^]^ and we adapt them as design guidelines for physical computers: That is, they can inform the physical configuration and actuation of the reservoir to improve computing performance.

Stepping further, using a robotic manipulator as a physical computer offers a natural pathway to applying its computing capacity to meaningful tasks. To this end, we equip the adaptive kernel with shape memory actuators and show that it could use its body dynamics to estimate the weight and orientation of the end payloads, as well as reconstruct the input command. This information can inform effective closed‐loop control. Overall, this study broadly proves that the mechanical design and input setup are closely related to physical computing performance. Given that the spectral and spatial correlations apply to any structures exhibiting multi‐degree‐of‐freedom dynamics, the outcome of this study could be used to make many other soft robots and multi‐functional materials more intelligent in the mechanical domain to meet demanding performance requirements.

## 2 Setting Up the Physical and Adaptive Computing Kernel

### 2.1 Origami‐Inspired Metastable Module Fabrication

**Figure 2:**
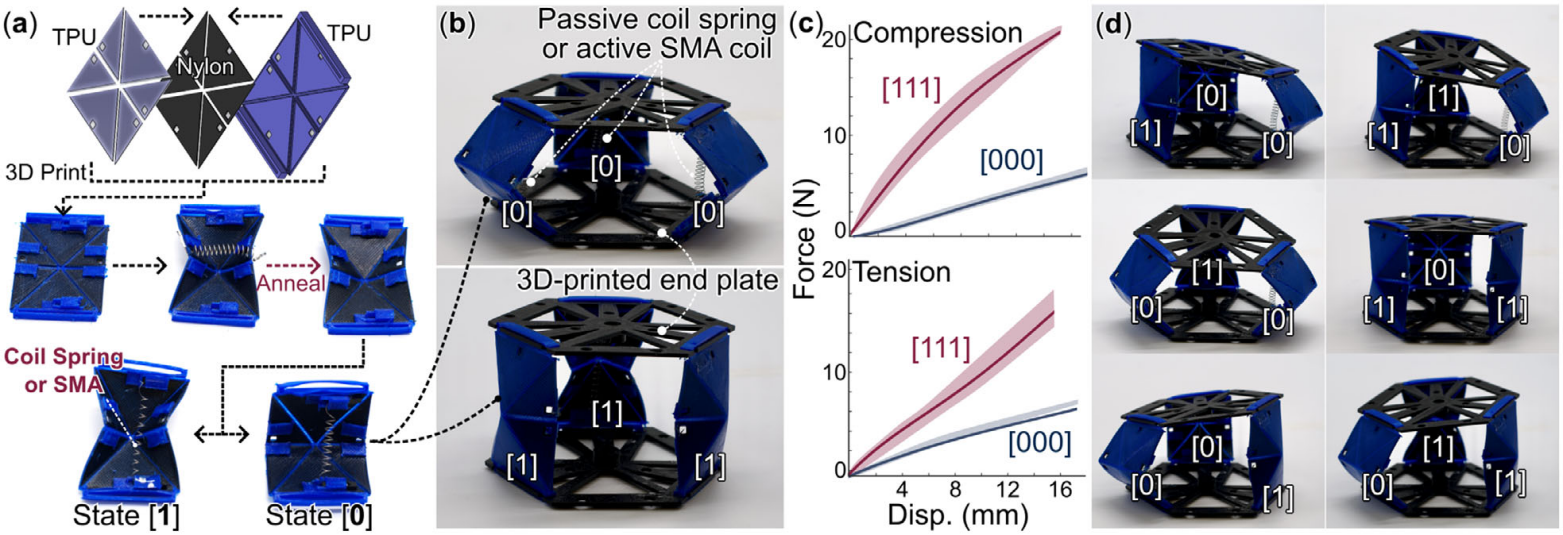
Fabrication and configuration of the origami‐inspired meta‐stable modules. a) To fabricate a bistable origami panel, we first 3D‐printed it using flexible TPU and stiffer Nylon filaments and heat‐treated it to reset its stress‐free folding configurations. After that, we fit the origami panel with a coil spring or an SMA actuator coil, giving it the desired bistability featuring soft [0] and stiff [1] states. b) We assembled three such panels and two 3D‐printed end plates to complete the meta‐stable module as the fundamental element in our adaptive physical computing kernel. Here, we focus on the most compliant [000] and stiff [111] states. c) Tensile and compression testing reveal that the longitudinal stiffness ratio between the [111] and [000] states is 4.00 ± 0.15. The solid lines are averaged test results from 10 loading cycles, and the shaded bands are the corresponding standard deviation. d) Close‐up view of the six other metastable states from the same module.

We design the robotic module to be lightweight, easy to fabricate, swappable, and with adjustable stiffness. This allows us to exploit its switchable properties to understand how physical reconfiguration influences computing capabilities. Each module comprises two 3D‐printed PLA base plates connected by three bistable origami panels (**Figure** **2**). These origami panels are based on the classic Yoshimura pattern^[^ ^39^ ^]^ and are 3D‐printed using a dual‐material FDM printer (Ultimaker S5). They feature a multi‐layer construction: A 0.4 mm thin base layer is first laid down using compliant TPU 95A filaments. Then, a middle layer of six 0.6 mm thin Nylon triangles is added to increase the facet stiffness, creating the desirable folding kinematics. Finally, a 0.2 mm thin TPU covers the whole panel to ensure proper bonding between different layers, even under large deformation. (More fabrication details in Appendix, Note S1, Figure S1, Supporting Information)

Once the 3D printing finishes, the origami panels are then manually folded inward along the creases, fixed by a coil spring, and then annealed at 110 °C for 10 min (Figure 2a). After the panels cool to room temperature, we remove the coil springs. This heat treatment eventually reset the stress‐free resting configurations of the origami panels from flat to folded, thus conditioning them to exhibit multi‐stability. Finally, we re‐attach the coil spring or a SMA coil actuator to the origami panel and create a bi‐stable assembly: a flexible state [0] and a stiff state [1] (notice the coil spring is now in a different orientation than the previous heat treatment step). Therefore, once the three origami panels are attached to the base plates to create a robotic module, it possesses eight (2^3^) different meta‐stable configurations (Figure 2d).

Once the meta‐stable modules are complete, we assemble them into a computing kernel simply using plastic screws through connection holes already printed in the base plates. The local stiffness and shape of these modules could be easily adapted by switching the origami panels between their [0] and [1] states. This study aims to examine the modular structure's adaptive computing capability, so we focus on the fully flexible [000] and the fully stiff [111] states. Compression and tension tests (Instron 6500 with 10 N load cell) show that the module in state [111] is approximately 4.00 ± 0.15 stiffer than in state [000] (Figure 2c).

### 2.2 Computation Tasks Overview

**Figure 3:**
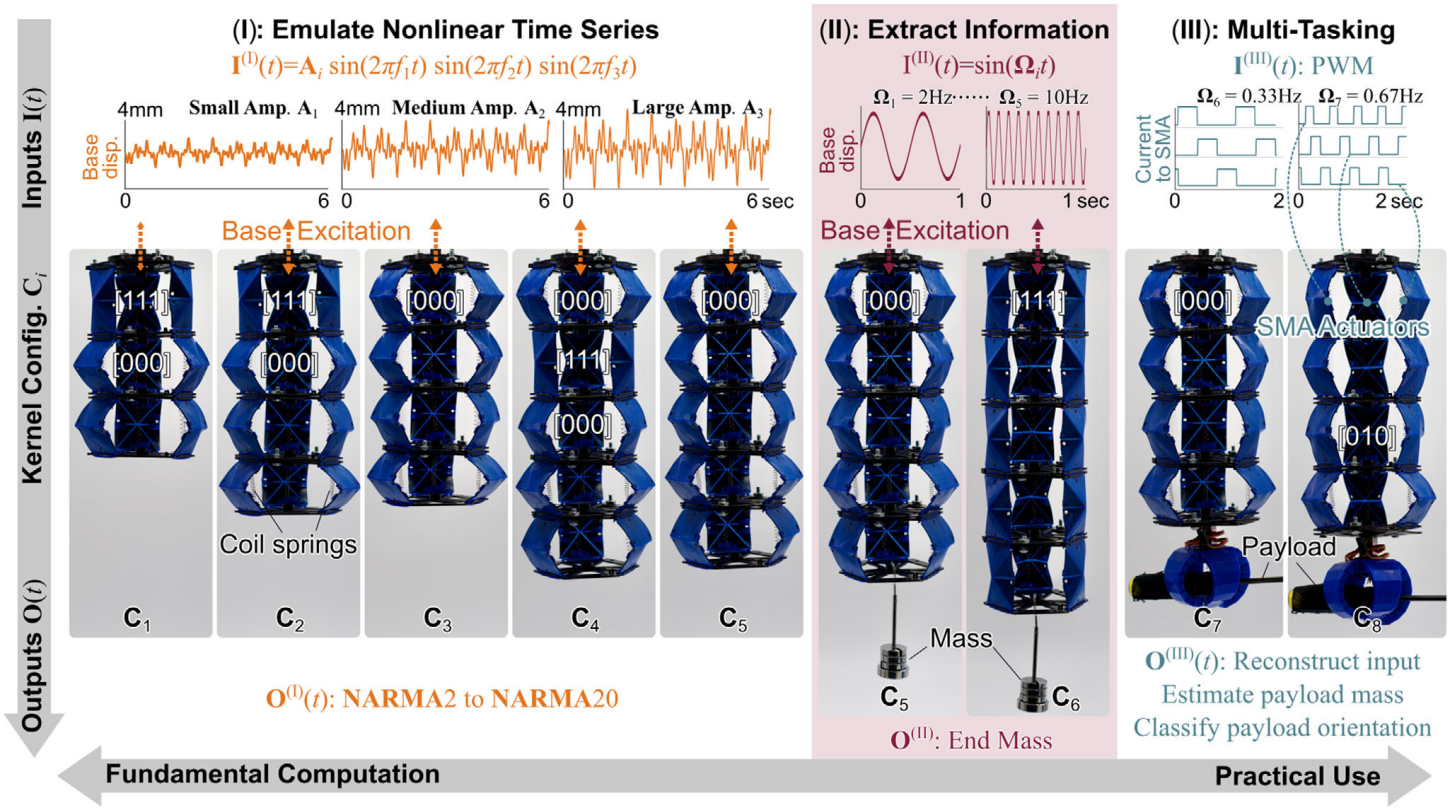
A summary of the computing tasks in this study, ranging from the more fundamental task (I) to the more practical multi‐tasking (III). We investigated these three tasks using different physical configurations (**C**_1_ to **C**_8_) by adapting the number and stable states of origami modules. In the time series emulation task (I), the input to the adaptive kernel is a base excitation **I** ^(I)^(*t*) consisting of three harmonic signals; and the targeted output is a time series defined by nonlinear NARMA equations. In the information extraction task (II), we attached masses to the adaptive kernel's free end. The input is a simple harmonic base excitation with different frequencies, and the targeted output is the weight of these end mass. In the final multi‐tasking (III), we replaced the embedded passive coil springs with active SMA coils and input Pulse Width Modulated (PWM) current to these SMA to excite (swing) the kernel. The output targets are to reconstruct the PWM input commands, classify the payload by estimating its weight, and determine the orientation of a payload.

In this study, we test the adaptive physical kernel's capability to complete three computing tasks, aiming to answer the big‐picture questions mentioned in the introduction. These tasks cover a broad spectrum from the fundamental computing capacity (i.e., “how much can the adaptive kernel compute?”) to the application of physical computing (i.e., “how useful is the adaptive kernel?”) (**Figure** **3**).

In the first Task (I), we challenge the adaptive kernel to complete the Normalized Auto‐Regressive Moving Average (NARMA) task. This benchmark task quantifies the computational capacity of a neural network based on nonlinear time‐series emulation.^[^ ^40^ ^]^ We test the adaptive kernel's NARMA performance using three base excitation magnitudes (**A**_1_, **A**_2_, **A**_3_) and five physical configurations (**C**_1_, …, **C**_5_) to dive deep into the correlations between the computing performance and structural setup.

In the second Task (II), we ask the adaptive kernel to estimate the weight of a random payload attached to its free end (Figure 3b). This task only involves two physical configurations (**C**_5_, **C**_6_), but five different base excitation input frequencies (**Ω**_1_, …, **Ω**_5_). This setup lets us understand how to extract the most computing capacity from a given physical kernel.

In the first two tasks, the adaptive kernel is attached to a large‐stroke shaker (APS 113) that provides the input signals via base excitation. In the third and final Task (III), we embed shape memory alloy (SMA) coil actuators to transform the adaptive kernel into a functional robotic arm and challenge this robotic kernel to estimate the weight and orientation of its payloads as well as to reconstruct the input commands. Here, proper input frequency settings (**Ω**_6_, **Ω**_7_) and physical configurations (**C**_7_, **C**_8_) are also crucial for achieving satisfactory performance.

Although the experimental setups and adaptive kernel configurations differ across these three tasks, the core computing framework is the same. The modular structure serves as the physical reservoir or mechanical neural network, mapping low‐dimensional dynamic input into a high‐dimensional state space, which is represented by the nodal (marker) displacements (*s*_*i*_(*t*)) along the structure. In this study, we measure these nodal displacements by processing the camera‐captured videos offline (Sonic a7C II camera with MATLAB image processing toolbox, Figure 1c). The computing output **O**(*t*) is simply a weighted linear summation of these state space vectors in that:

(1) $$O\left(t\right)=w_{0}+\sum_{i=1}^{n}w_{i}s_{i}\left(t\right),$$

where *s*_*i*_(*t*) is the displacement of the *i*_*th*_ node, and *w*_*i*_ is the readout weight (in this study, *n* = 40). The core principle of reservoir computing states that, with proper training, one can find an optimal set of readout weights so that the reservoir output matches the targeted output $O\left(t\right)\approx \hat{y}\left(t\right)$, thus completing a machine learning task.

## 3 Results and Insights

### 3.1 Task (I): Adapting the Physical Kernel for Optimal Time Series Emulation

This first task aims to uncover how to configure our adaptive kernel to maximize its computing performance. To this end, we use the widely adopted NARMA emulation task (The task is detailed explained in Appendix, Note S2, Supporting Information) to benchmark the computing output.^[^ ^40^ ^]^ In the experiment, the modular structure is attached to a vertically vibrating, long‐stroke shaker that provides the input signal as the base excitation (Detailed experiment setup in Appendix, Note S2, Figure S2, Supporting Information). This input signal is a product of three harmonic streams and an amplitude scaling factor *A*_*n*_:

(2) $$I_{i}^{\left(I\right)}\left(t\right)=A_{i}\sin2\pi f_{1}t\sin2\pi f_{2}t\sin2\pi f_{3}t.$$

**Figure 4:**
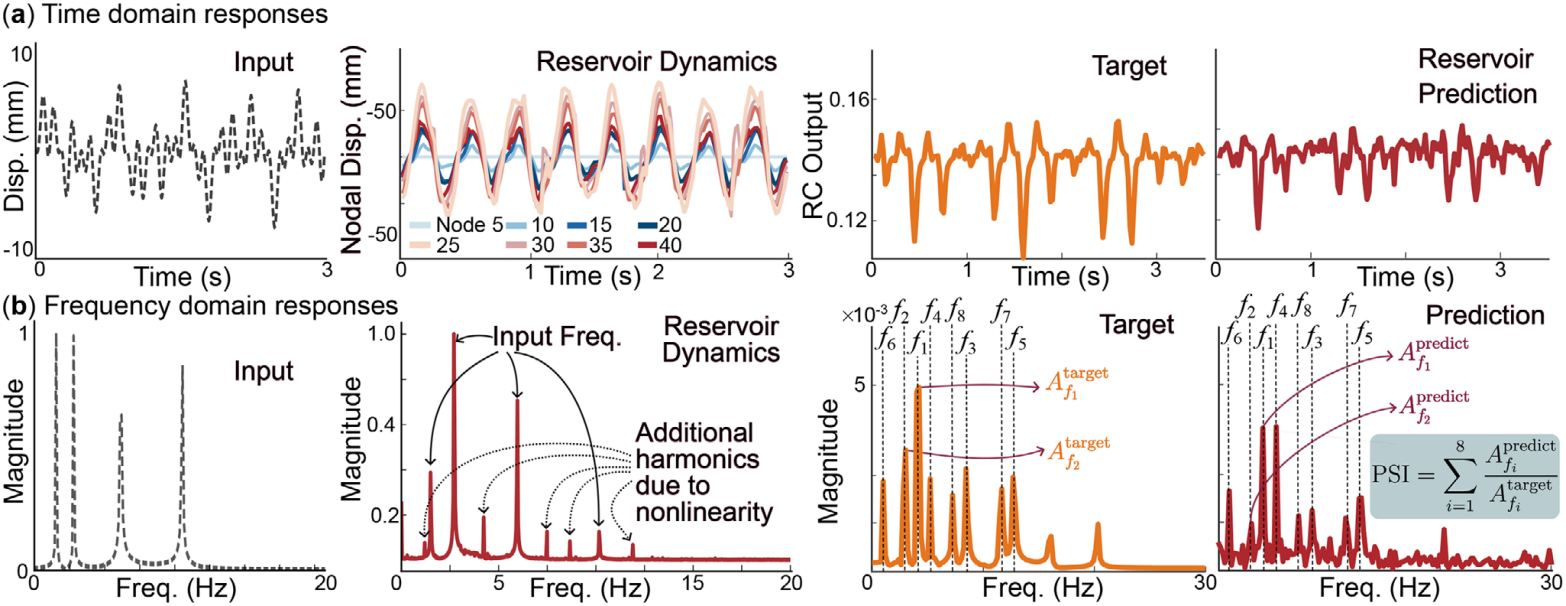
Temporal and spectral data used in the NARMA tests, as well as the definition of Peak Similarity Index (PSI): Here, we use the NARMA10 test results on Configuration 5 and Amplitude 3 (**C**_5_ **A**_3_) as the example: a) The time domain data of the input signal, reservoir dynamics, NARMA target function, and the corresponding reservoir outcome. b) The corresponding frequency domain data. Here, the spectral reservoir dynamics is the average of all nodes. The last figure illustrates how PSI is calculated based on the target spectral content and RC output.

The frequencies of the three harmonics are 2.11, 3.73, and 4.33Hz, respectively. All nodal displacements (or marker displacements) *s*_*i*_(*t*) are captured by a camera at 60 frames per second and extracted using the MATLAB image processing toolbox. **Figure** **4**a gives examples of these displacement fields.

Essentially, the nonlinear mapping between a one‐dimensional input stream **I** ^(I)^(*t*) and the corresponding displacement fields *s*_*i*_(*t*) captures the complex dynamics of the adaptive kernel. If this dynamics is “rich” enough, it can emulate the temporal response of a high‐order nonlinear dynamic system defined by the NARMA task. In other words, via proper training at the output readout layer (explained in Appendix, Note S2, Figure S3, Supporting Information), one can find a set of readout weights *w*_*i*_ so that the adaptive kernel's output **O** ^(I)^(*t*)(= *w*_0_ + ∑*w*_*i*_ *s*_*i*_(*t*)) can emulate the response of a nonlinear system $\hat{y}\left(t\right)$ in that

(3) $$\begin{array}{ccc} O^{\left(I\right)}\left(t\right)\approx \hat{y}\left(t\right),\;\text{where}\;\hat{y}\left(t+1\right) & = & \alpha \hat{y}\left(t\right)+\left(5\beta \hat{y}\left(t\right)\sum_{j=0}^{N-1}I^{\left(I\right)}\left(t-j\right)\right)\\ & & +\gamma I^{\left(I\right)}\left(t-N+1\right)I^{\left(I\right)}\left(t\right)+\delta , \end{array}$$

Here, the parameter *N* describes the complexity of targeted nonlinearity and, thus, the difficulty levels of the NARMA emulation task. Generally speaking, a reservoir that can finish higher‐order NARMA tasks can accomplish more sophisticated machine learning.^[^ ^40^ ^]^ This study focuses on *N* = 2, …, 20.

The input and output Equations (2) and (3) indicate that the NARMA task challenges the adaptive kernel to generate higher‐order harmonics output from a relatively simple input. For example, Figure 4b presents the spectrum of the input signal alongside that of the NARMA10 task. The substantial difference in spectral content between the input and NARMA10 target demonstrates that solving this task requires substantial nonlinear processing. In other words, additional frequency contents need to be generated from the reservoir dynamics. This behavior is very similar to “super‐harmonics,” a well‐studied concept in nonlinear structural vibrations. Such a parallel offers us a unique opportunity to adopt knowledge from the discipline of structural vibration to physical reservoir computing here. For example, most vibration experiments concluded that generating strongly nonlinear super‐harmonic dynamics requires two conditions. One is the intrinsic mechanical property: the structure must exhibit nonlinear and non‐uniform stiffness. The other is extrinsic input: the excitation magnitude must be large enough to evoke such nonlinearity. Figure 4b shows the presence of many additional peaks in the reservoir (*C*_5_) dynamics response that are not present in the input spectrum, confirming the nonlinear transformation capabilities of the system. Full spectra from all 15 physical settings (5 configurations × 3 amplitudes) are provided in Appendix, Figure S4 (Supporting Information).

Therefore, we adjust two types of “physical hyper‐parameters” in this study to explore their relationship with computing capability. The first type corresponds to the intrinsic mechanical property, which includes the number of origami modules and their stable states. These factors dictate the physical dimensionality and nonlinearity. More specifically, we experimented with five different physical configurations with varying combinations of flexible and stiff modules. They are labeled as **C_1_** to **C_5_** as shown in Figure 3. The second type of physical hyperparameter is the extrinsic input amplitude, and we used the shaker controller to input three levels of base excitation magnitude, labeled as **A_1_**, **A_2_**, **A_3_** as defined in Equation (2). In each computing experiment, the overall reservoir setting is labeled as **C_m_A_n_**, where *m* represents the physical configuration and *n* is the input magnitude scale (e.g., **C_1_A_1_** means configuration 1, excited by the input magnitude 1).

**Figure 5:**
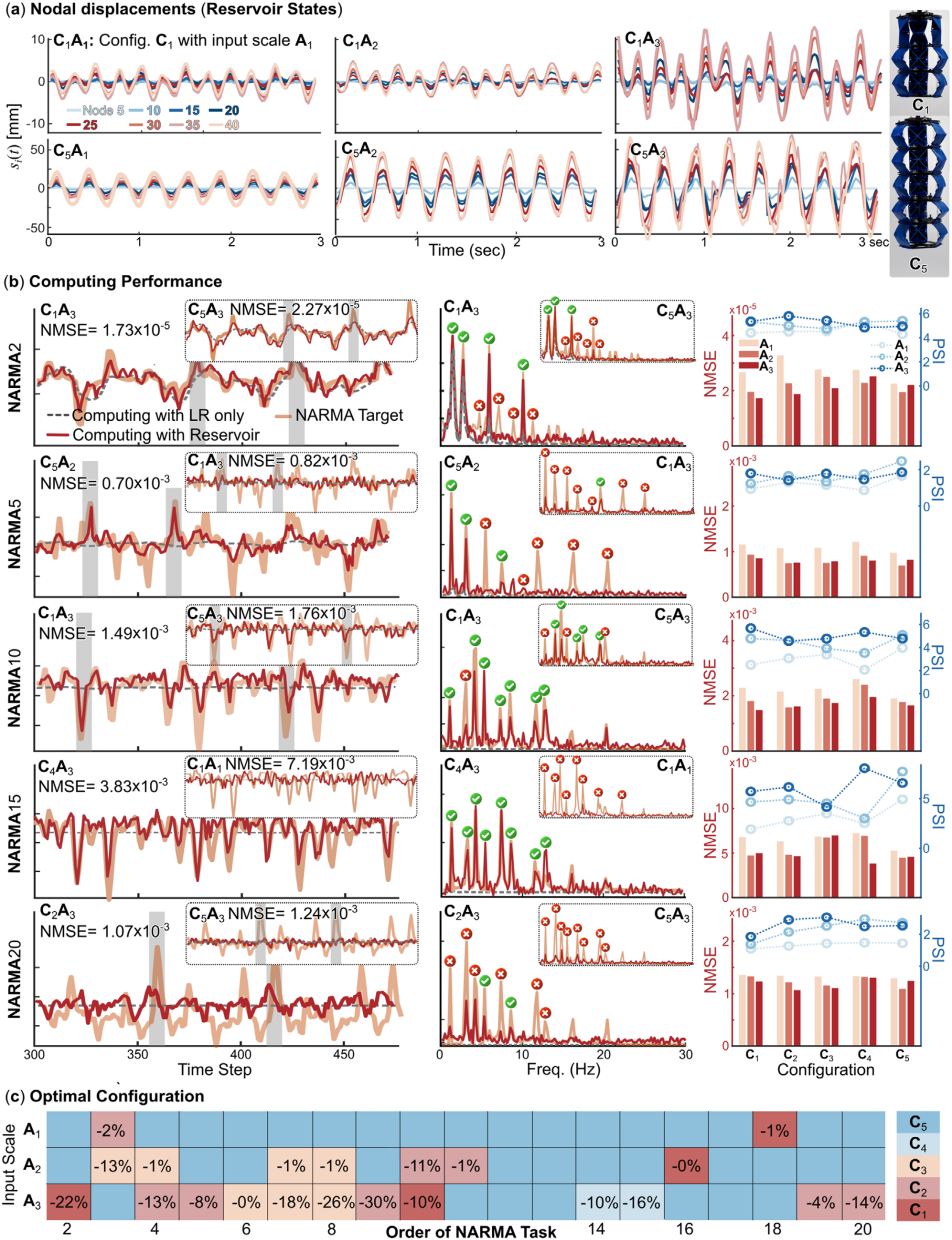
The adaptive kernel's computing performance using the NARMA benchmark. The reservoir dynamics differ, with different physical configurations **C**_*m*_ and base excitation magnitude **A**_*n*_. a) The nodal displacements of **C**_1_ and **C**_5_ under different input magnitudes. b) From left to right columns: The temporal outcome, spectral comparison, and NMSE/PSI summary for NARMA2, NARMA5, NARMA10, NARMA15, and NARMA20 tasks. (Note the vertical axes are not labeled here for visual clarity. It is the *similarity (or the lack of it)* between the NARMA targets and the reservoir output that matters.) c) The adaptive kernel's optimal configuration corresponds to different NARMA tasks and input magnitudes. The values on this color map are the percentage reduction of NMSE error compared to the **C**_5_ configuration performance at that input magnitude.

The first column of **Figure** **5**b summarizes the best temporal outcome from NARMA2, 5, 10, 15, and 20 tests and their corresponding **C_m_A_n_** setup. For comparison, the smaller inserts at the upper‐right corner show another outcome from a different, randomly selected setting. It is evident that, with the correct combination of physical configuration and input magnitude, the modular reservoir can closely emulate the targeted dynamics (Movie S1, Supporting Information, shows diversified NARMA Emulation performance with different physical configurations), even if the NARMA tasks ask for very high‐order and complex features like the shaded area marked in Figure 5b. To quantify the NARMA computing performance, we adopt the **N**ormalized **M**ean **S**quare **E**rror (**NMSE**) between the reservoir and target outcomes (NMSE is defined in Appendix, Note S2, Supporting Information). The errors from all computing experiments are summarized in the third column of Figure 5(b). Interestingly, our adaptive reservoir outperforms a prior work using a significantly softer, silicon‐based robotic arm as explained in Appendix, Note S3, Table S1 (Supporting Information) indicating that a softer structure does not always lead to better reservoir computing outcomes. While soft materials generally show material nonlinearity that is naturally advantageous for reservoir computing, our origami reservoir—though made from relatively stiff materials—demonstrates strong geometric nonlinearity caused by features like bistability and finite rotations of origami folds and creases. This supports a broader perspective that the effectiveness of a mechanical reservoir should not be inferred solely by its softness (or the lack of it), but by its capacity to produce diverse, high‐dimensional, and nonlinear dynamic responses.

Another lesson from the discipline of nonlinear structural vibration is that *spectral* analysis is as informative, if not more important, than the temporal response. Therefore, the second column of Figure 5b shows the corresponding spectral content via fast Fourier analysis, which offers a much deeper insight into the computing performance of our adaptive kernel. More specifically, the targeted NARMA output contains several harmonic peaks: Some are at much higher frequencies than the input frequencies, and more difficult NARMA tests generally have more peaks. The adaptive kernel performs better if it can replicate these peaks via its inherent nonlinearity and superharmonic behaviors. In the spectral plot, we use green check marks to indicate a successfully reproduced peak and red cross marks to indicate a harmonic peak missed by the reservoir.

Therefore, we define a **P**eak **S**imilarity **I**ndex (**PSI**) to quantify the NARMA task performance in the spectral domain. The PSI is the ratio of the reservoir output's spectral peak magnitudes over the corresponding targeted peak magnitude based on NARMA, as presented in Figure 4b. In our paper, we focus on the first eight primary harmonic peaks. Therefore, the maximum PSI value is 8, meaning the adaptive kernel perfectly reproduces the first eight harmonic peaks required by the NARMA task. The minimum PSI is 0, meaning the reservoir fails to generate any desired harmonics (more details on PSI are in Appendix, Note S2, Figures S4 and S5, Supporting Information). The third column of Figure 5b summarizes the PSI from all computing tests, and the inverse correlation between Mean Square Error and Peak Similarity Index is evident. A correlation analysis between PSI and NMSE across all five NARMA tasks is included in Appendix, Note S2, Table S1 (Supporting Information) to prove a strong negative relationship between PSI and NMSE.

#### Lessons Learned

We can arrive at several intriguing conclusions by carefully assessing and comparing the computing performance from different physical settings. First, a larger input magnitude helps to achieve better computing performance. The temporal error is lower (and spectral similarity is higher) when the input magnitude scale is larger at **A**_3_. This trend is particularly evident in the more difficult NARMA tests. The more difficult NARMA demands significantly more nonlinear dynamics (e.g., NARMA15 requires more harmonic spectral peaks than NARMA2). A larger input excitation can evoke such rich dynamics from the intrinsic mechanical nonlinearity.

Second and most surprisingly, there is no optimal physical configuration (aka. there is no “one size fits all” solution to physical computer design, Figure 5d). **C**_5_ is generally the favored configuration at a smaller input magnitude **A**_1_, likely because the smaller input does not invoke a significantly nonlinear response, and the additional modules in the **C**_5_ configuration can compensate for such a lack of nonlinearity. However, at the higher input **A**_3_ (with better overall NARMA scores), the optimal configuration changes between different NARMA tasks without apparent trends.

These observations highlight the advantages of making the physical computer adaptive and modular. As different computing tasks can have widely different requirements, an adaptive physical kernel that can intentionally and intelligently reconfigure its physical architecture has a much broader chance of success.

Finally, we conducted an additional experiment that only involves reconfiguring a 5‐module adaptive kernel. The outcome of this set of experiment are summarized in Appendix, Note S4, Figures S6– S8 (Supporting Information). The lessons above still apply.

### 3.2 Task (II): Extracting Payload Weight Information with Minimal Training Data

While the previous task aims at fundamental computing capacity, this task focuses more on leveraging such computing power for more practical uses, like information perception and robotic control. In particular, we challenge the adaptive kernel to output the weight of a payload attached to its free end using the information encoded in its dynamic responses and minimal training data (Figure 3).

Unlike the NARMA tests that require strong nonlinearity, payload weight estimation is closer to a linear classification task. By exploiting our physical kernel's modular and adaptive nature, we can use this task to answer another “big‐picture” question regarding physical computing: Once the physical design is fixed, how can we use minimal training data to achieve optimal computing performance? Ideally, the required training data is lower if the mapping from the payload to structural dynamics is a linear transformation, but a pure linear transformation might not be robust against the noises and uncertainties in the practical implementations.^[^ ^41^ ^]^

To this end, we tested two configurations, **C**_5_ and **C**_6_, with five modules all in the compliant state [000] or all in the stiff state [111], respectively. The shaker input, in this case, is a simple harmonic stream **I** ^(II)^(*t*) = *A*sin **Ω** *t*, where the harmonic frequency ranges from **Ω**_1_ = 2 to **Ω**_5_ = 10 Hz (Figure 3). The targeted output of this task is simply the payload weight, which has five distinct values: 0 grams (i.e., no payload), 50, 90, 130, and 170 grams. The readout weights *w*_*i*_ are unique for each combination of input frequency and payload weight setting and must be trained separately (detailed readout weight training procedures are in the Appendix, Note S5, Supporting Information).

**Figure 6:**
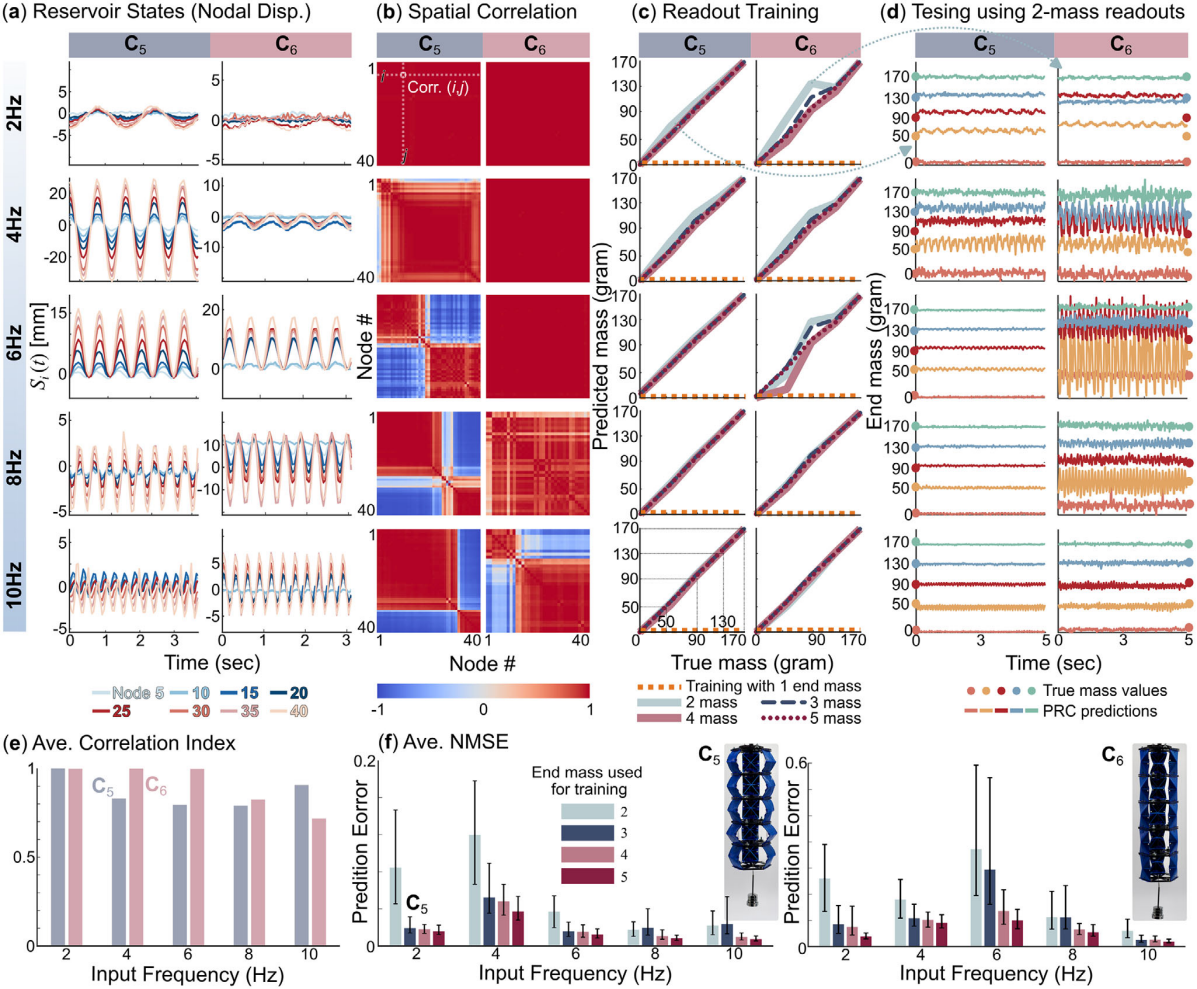
Extracting payload weight from body dynamics, using different kernel configurations and input conditions. a) The adaptive kernel's body dynamics are represented by nodal displacements. b) The correlation matrix between nodal displacements provides a quantitative measure of the “richness” of a physical body's dynamic characteristics. c) The adaptive kernel estimates its payload mass using different amounts of readout training data. If one uses the data from all five payloads for readout training, the kernel can output very accurate weight estimation. On the other hand, if one only uses the data from 1 payload, the kernel will fail the estimation task. d) The reservoir's prediction using readout weights trained using data from two payloads. These results are accurate only when the spatial correlation between nodal displacements drops. e) Averaged spatial correlation between different physical and input settings. f) The overall estimation error in all testing cases with different readout training setups.

First, we excite the adaptive kernel without payload at different base excitation frequencies to understand its fundamental dynamics. The corresponding state vectors *s*_*i*_(*t*) are recorded by the camera and summarized in **Figure** **6**a. Here, we borrow another helpful concept from the structural vibration discipline: spatial correlation, which describes the similarity between the time responses from two points of a structure (explained in Appendix, Note S5, Supporting Information). A correlation of 1 means two signals are nearly the same, and a correlation of –1 implies the opposite. Figure 6b summarizes the correlation between the different nodal displacements from Figure 6a. One can observe a “threshold” frequency, above which the correlation dropped significantly, meaning that the adaptive kernel's body dynamics become diverse and complex (Movie S2, Supporting Information). The magnitude of such a threshold frequency is inherently related to the intrinsic stiffness. The nodal displacement correlation started dropping above 4Hz with the softer **C**_5_ configuration and above 8 Hz input with the stiffer **C**_8_ configuration. This difference indicates that the correlation is directly related to the structure's primary resonance frequency.

Then, we added payload masses to see if the adaptive reservoir could exploit its dynamics to estimate the weights. For each physical setup **C**_*m*_ **Ω**_*n*_, we attach the four masses one after another, thus obtaining five sets of temporal response data. This setup allows us to experiment using different amounts of data for readout training. For example, we start by using 5 s of the nodal displacement data with two payloads (0 and 170 grams) for readout training. That is, denote *S*_0_(*t*) and *S*_170_(*t*) as the reservoir state space from 0 and 170‐gram payloads, respectively. We can combine these two state spaces into a larger set *S*(*t*) = [*S*_0_(*t*) *S*_170_(*t*)], and use the corresponding payload weight to define a step‐wise function as the targeted output:

(4) $$\hat{y}\left(t\right)=\left\{\begin{array}{cc} 0\; & 0\leq t\leq 5\\ 170\; & 5\leq t\leq 10 \end{array}\right.$$

The readout weights *w*_*i*_ can be obtained via linear regression so that the adaptive kernel's output $O^{\left(\text{II}\right)}\left(t\right)=w_{i}S\left(t\right)\approx \hat{y}\left(t\right)$. Then, we apply this set of readout weights to the nodal displacements with other payloads to obtain a prediction of their weights. The results are summarized in Figure 6d. With low input frequencies, when the structural dynamic correlation is high, the adaptive kernel performs poorly with a significant estimation error and oscillatory, unstable output. On the other hand, when the correlation matrix between nodal displacements starts to drop (or the body dynamics start to become richer and more complex), the modular kernel performs significantly better.

One can always use more data for readout training to improve prediction accuracy. Figure 6c summarizes the adaptive kernel's prediction using 1 to 5 sets of nodal displacement for readout training (Detail training with different pair of data is explained in Appendix, Note S5, Figure S9 (Supporting Information). Appendix, Movie S2 (Supporting Information) shows varied weight estimation performance with different physical configuration and training methods). Suppose we use only one set of nodal displacement data (e.g., corresponding to 0‐gram payload) to train a readout and apply this readout to the nodal displacements with other payloads. The adaptive kernel would fail the weight estimation task (dashed orange curves in Figure 6c). On the other end, we can achieve the best prediction by using all data with the five payloads for the readout training (red markers in Figure 6c). This way, the prediction error is always lower than 2% in the flexible **C**_5_ configuration and 10% for the stiffer **C**_6_, regardless of the input frequency (dotted red curves in Figure 6c). However, this is inefficient and wastes computing resources.

#### Lessons Learned

Our comparative tests show that complex and diverse body dynamics—indicated by a low spatial correlation between different nodes—are critical for reducing the required training data. Once the correlation is sufficiently low, one only needs the response data with two payloads for readout training. To reduce the correlation, one can reconfigure the adaptive kernel to lower its intrinsic stiffness (or resonance frequency).

### 3.3 Task (III): Robotic Multi‐Tasking with SMA‐Activation

**Figure 7:**
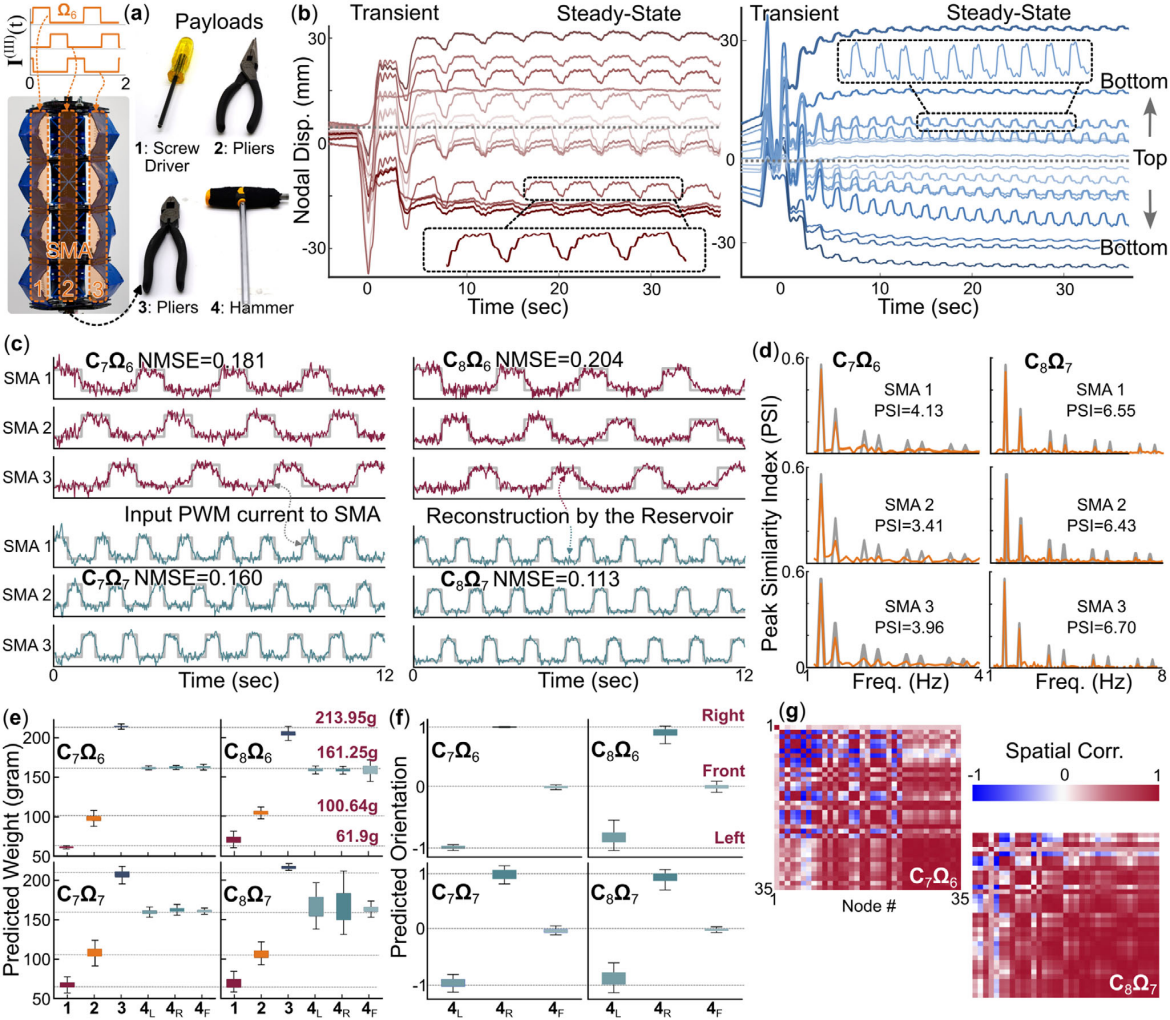
Robotic multi‐tasking (III). a) The embedded shape memory alloy (SMA) coil actuator setup, the PWM input voltage command, and the payloads used in this study. b) The nodal displacements under two different input frequencies. (For visual clarity, the vertical axes are not labeled in here. It is the *similarity (or lack of it)* between the actual SMA input and the reservoir predictions that matters.) c) The reconstructed SMA input commands from the adaptive reservoir based on four different setups. d) The PSI of two example cases: **C**_7_ **Ω**_6_ and **C**_8_ **Ω**_7_. Similar to Task (I), higher PSI gives a better time‐series emulation performance. e) Payload weight estimation results, and (f) hammer orientation classification results. g) Correlation matrix between different nodal displacements of the two example cases. Like Task (II), lower spatial correlation results in better information extraction performance.

In the final task, we explore the potential of applying embodied physical computing for realistic robotic functions. To this end, we replace the passive coil springs with active shape memory alloy (SMA) coils in the origami modules, making the adaptive kernel into a robotic manipulator. These SMAs can provide robotic actuation for object manipulation and structural support. Therefore, we attached different machine shop tools to the modular arm's free end as the payload, including a screwdriver, a hammer, and two pliers with different weights (**Figure** **7**a; Appendix, Figure S10, Supporting Information). Here, the hammer has a significantly eccentric center of mass, which can substantially influence the manipulation outcome, so knowing its orientation is also helpful. Therefore, we challenge the robotic kernel to achieve three computing sub‐tasks.

The first sub‐task is to emulate (or reconstruct) the input electric command to the SMA coils. This is a time‐series emulation task similar to the NARMA Task (I); however, in this case, the targeted outcome $\hat{y}\left(t\right)$ is the voltage input to the SMA actuator in that $\hat{y}=I^{\text{(III)}}\left(t\right)$ as illustrated in Appendix, Figure S12 (Supporting Information) The second sub‐task is to classify the end payloads by estimating their weights. If the end payload is the hammer, the robotic kernel must complete the third sub‐task: differentiating the hammer's orientation. These two sub‐tasks are similar to the payload weight estimation Task (II). It is worth highlighting that these three sub‐tasks use the same nodal displacement data, so we are essentially re‐purposing the robotic kernel into a multi‐model sensor that simultaneously extracts complex information to inform effective control.

We grouped the SMA coils into three columns and connected those within each column in series. This way, we can activate each column of SMA with a **P**ulse **W**idth **M**odulated voltage input (**PWM**), labeled as $I_{1}^{\left(\text{III}\right)}$, $I_{2}^{\left(\text{III}\right)}$, and $I_{3}^{\left(\text{III}\right)}$ respectively (Figure 7a). Such input can bend the robotic kernel for end payload manipulation while simultaneously generating the dynamic response required for reservoir computing. The mechatronic design for SMAs actuation is illustrated in Appendix, Note S6, Figure S11 (Supporting Information).

To investigate the influence of different dynamics on this task performance, we set the adaptive kernel into two configurations: **C**_7_ and **C**_8_. In **C**_7_, all modules are configured in the entirely soft [000] state. Meanwhile, in **C**_8_, the modules are in the [010] state, which creates asymmetric stiffness in the robotic arm so that it would bend even under uniform SMA actuation. In addition, we combined these two configurations with two PWM input patterns: **Ω**_6_ and **Ω**_7_. **Ω**_6_ would joule heat the SMA coils for 1 s, followed by 2 s of cooling and relaxation, giving a 0.33Hz actuation frequency. Meanwhile, **Ω**_7_ is 0.67Hz, giving 0.5 s of heating and 1 s of cooling (Figure 3). Consequently, we test four physical settings: **C**_7_ **Ω**_6_, **C**_7_ **Ω**_7_, **C**_8_ **Ω**6, and **C**_8_ **Ω**_7_, as shown in Figure 7.

Regardless of the kernel configuration and PWM input setup, the modular robotic arm would exhibit random and significant transient dynamics due to the local buckling of some origami panels. After a few seconds, it settles into a steady state and small amplitude swing motion around an equilibrium shape (Figure 7b). Such swinging motions, caused by alternating actuation of the three columns of SMA coils, are recorded by the camera and serve as the training and testing dataset. Appendix, Figures S12 and S14 (Supporting Information) details the training and testing procedures for input emulation task and payload weight/orientation identification tasks, respectively.

Figure 7c shows the performance of the input command emulation tasks across different physical settings (Detailed error analysis is detailed in Appendix, Figure S13, Supporting Information). The modular kernel's output can emulate (or reconstruct) the input command with reasonably high accuracy. In particular, configuration **C**_8_ **Ω**_7_ achieved the best emulation performance. Moreover, higher‐frequency actuation provides better input signal mapping. The increased stiffness and higher actuation frequencies enhanced the robotic arm's dynamic synchronizing with the input command, leading to a higher spectral correlation between the input and output (i.e., higher peak similarity index, as shown in Figure 7d).

Figure 7e summarizes the results of payload weight estimation under the four physical settings. In all cases, reservoir outputs are well separated across repeated experiments, enabling clear differentiation among the four payloads. Interestingly, the optimal physical settings for payload estimation differ from those for input command emulation. In this case, the more flexible configuration (**C**_7_) exhibits more consistent performance with more minor errors away from the ground truth (MSE lower than 5%). The prediction error is also significantly minor under the lower actuation frequency **Ω**_6_. These observations suggest that the robotic kernel exhibits richer body dynamics under these conditions, exhibiting a lower spatial correlation between different nodal displacements (Figure 7g).

Finally, suppose the end payload is identified as the hammer. In that case, we apply another set of trained readouts to the same set of nodal displacement data to predict its orientation (i.e., the hammerhead toward the left, right, or front). Figure 7f clearly illustrates that all physical settings allow the robotic arm kernel to distinguish the hammer's orientation. Appendix, Movie S3 (Supporting Information) demonstrates the How manipulator uses multi‐tasking capability to reconstruct three input commands and identify accurate payload information in parallel with collected dynamics.

#### Lessons Learned

First, this multi‐tasking experiment exemplifies the potential of embodied physical computing in soft robotic systems. By swinging the robotic arm using embedded SMA actuators, we can generate rich body dynamics that contain useful input commands and payload information. Physical reservoir computing is an efficient way to extract this information.

Moreover, the results of this multi‐tasking study point to a trade‐off between different computing and information perception tasks. The optimal physical configuration and input setups depend on the computing task's nature. Flexible structures with lower actuation frequencies improve information extraction outcomes (i.e., classifying the payload weight and orientation). In comparison, stiffer configurations with higher actuation frequencies enhance time series emulation (i.e., input command reconstruction). This insight again highlights the benefit of using adaptive physical kernels for different embodied computation tasks so that one can reconfigure them accordingly.

## 4 Discussion and Conclusion

First, it is worth emphasizing that our adaptive kernel is repurposed from a fully functional soft robotic arm and is never specially designed for physical reservoir computing. Regardless, it shows impressive computing and machine‐learning capability in the mechanical domain. In particular, this robotic arm's meta‐stable and modular nature allows us to uncover the connection between physical configuration and computing performance. Therefore, the results of this study can be applied to many other reconfigurable soft robotic systems, revealing their embodied computing power without compromising (or even enhancing) their original robotic capabilities.

Secondly, because our adaptive kernel is repurposed from a robotic arm, we choose to use simple markers with image processing to extract its body dynamics. In this way, we do not need to add any mechatronic components to the robotic arm. Additionally, to facilitate consistent comparison across all tasks and setups, nodal displacement is selected as the common and accessible method to represent system dynamics. However, it should be noted that the system's dynamic behavior can be represented by other more complex physical variables, such as angular variations, local material bending, and twisting. Thus, in a parallel proof‐of‐concept study, we show that it is also possible to add embedded sensors like strain gauges to measure body dynamics without a camera. In this way, one can achieve fully online physical computing and information extraction.^[^ ^42^ ^]^

Finally, to consolidate and synthesize the lessons learned across tasks into more generalizable design principles, we highlight the following observations and present them as the answers to the big‐picture research questions from the beginning:

1. *Integrating physical adaptation and input control is an effective strategy to ensure robust nonlinear dynamics for reservoir computing*. In this study, we used a modular setup and multi‐stability to achieve physical adaptation, and we adjusted the base excitation frequency and amplitude to control the input. This approach allows us to evoke a broader range of dynamics with a wider spectral content for different computing tasks. Indeed, physical adaptation and input control can take various forms in other reservoir computers—for example, physical adaptation may involve changes in a material's crystal structure^[^ ^43^ ^]^ or growth of living neurons^[^ ^44^ ^]^—but combining these two elements systematically can significantly enhance the reservoir computing outputs.
2. *There is no universal reservoir design—task‐specific optimization is essential, and physical adaptability is crucial for general‐purpose physical reservoir computers*. In this study, we demonstrated that spectral correlation (PSI) and spatial correlations can effectively evaluate the alignment between physical properties and task requirements. These two correlation indices, by definition, are design‐agnostic and apply to any physical system with multiple degrees of freedom and nonlinear dynamics, making them useful for various physical reservoir computing setups.
3. *Lower spatial correlation (i.e., greater spatial diversity of dynamics across the structure) decreases the need for large training datasets*. This insight can guide practical implementations of physical reservoir computing for truly online and real‐time computation with minimal readout and output layer overhead.

Note that these observations do not strictly correspond to the three big‐picture questions 1‐to‐1. Instead, combining them reinforces a core theme of this work: effective physical reservoir design should not focus solely on material properties or morphology in isolation, but rather on how the physical system transforms itself and projects input signals into dynamic responses that are spectrally and spatially well‐aligned with the targeted computation tasks. By merging materials science, robotics, and computational intelligence, this work lays the foundation for next‐generation embodied AI systems, where robots use their physical structures as an intrinsic part of intelligent computation and control.

## Conflict of Interest

The authors declare no conflict of interest.

## Supporting information

Supporting Information

Supplemental movie 1

Supplemental movie 2

Supplemental movie 3

## Data Availability

The data that support the findings of this study are available in the supplementary material of this article.
