# Supplementary material for: Re‐Purposing a Modular Origami Manipulator Into an Adaptive Physical Computer for Machine Learning and Robotic Perception
Source: Adv Sci (Weinh). 2025 Sep 14;12(45):e09389. doi: 10.1002/advs.202509389 (PMC12677637; doi:10.1002/advs.202509389)
Supplement: Supplementary file 1 — Supporting Information [file ADVS-12-e09389-s004.pdf]

# Supporting Information for

## Re-purposing a modular origami manipulator into an adaptive physical computer for machine learning and robotic perception

**Jun Wang and Suyi Li**

**Jun Wang.**
**E-mail: junw@vt.edu**

**This PDF file includes:**

Supporting text
Figs. S1 to S18
Table S1
Legends for Movies S1 to S3
SI References

**Other supporting materials for this manuscript include the following:**

Movies S1 to S3

## Supporting Information Text

### Note S1: Origami Module Fabrication and Assembly.

***CAD Modeling.*** The 3D CAD model of the origami panel and the stiff connecting base plates is developed using SolidWorks 2024, as shown in **Figure S1**. The classical Yoshimura pattern inspires the crease design of the bistable origami panel—A rectangular shell composed of six triangular facets. The panel comprises three layers: a 0.4mm thermoplastic polyurethane (TPU) 95A base layer, a 0.6mm stiff Nylon layer forming the six triangular facets, and a 0.2mm thin TPU top layer that covers and bonds the Nylon to the base. Nylon is selected as the printing material for the middle layer for its high stiffness, significantly enhancing the panel's metastability. TPU 95A primarily serves as the crease material, ensuring sufficient flexibility for folding and reconfiguration.

Figure S1(a) illustrates the top, side, and front views of the assembled origami panel. Several design modifications were made to adapt the structure for fabrication, distinguishing it from conventional paper-based Yoshimura models. First, a gap consisting solely of the TPU base layer (blue components in Figure S1a) is introduced between the thick triangular facets (black regions formed by the TPU-Nylon-TPU sandwich structure) to enable proper folding, as it maintains negligible thickness and sharp fold lines. Second, three additional components are integrated to facilitate manufacturing. Hollow TPU cubes located at the top and bottom of the panel enable simple mechanical connection to the rigid plate by press-fitting into panel-base connectors positioned at the plate's edge (Figure S1b). Additionally, two TPU-printed housings are included to accommodate spring coils or shape memory alloy (SMA) actuators. Four square cutouts along the centerline are designated for placing the coil spring during the annealing process. The rigid base plate is designed as a 1mm-thick hollow hexagon, with panel-base connectors cut into three of its edges to allow simple attachment of the origami panels. Additionally, nine circular cutouts are incorporated into the plate to facilitate inter-module connections using screws.

***3D Printing.*** In this study, we employed the widely used fused deposition modeling (FDM) technique to fabricate the origami panel and rigid base plate using a dual-material Ultimaker S5 3D printer. A layer-by-layer dual-material printing strategy was adopted for the origami panel following the methodology presented in our previous work (1).

To ensure acceptable printing resolution, the layer thickness for 3D printing was set to 0.1mm. First, the TPU 95A base layer (0.4mm thin) was printed using a zig-zag path and a printing core temperature of 225°C, providing a durable and flexible foundation. The Nylon triangular facets were then printed on top using a zig-zag pattern, with the core temperature set to 250°. Finally, a 0.2mm thin TPU top layer, printed under the same conditions as the base layer, was deposited to encapsulate and firmly bond the Nylon layer, ensuring structural integrity under large mechanical stress and deformation. The rigid base plate was printed solely with PLA material using a zig-zag infill pattern at a core temperature of 205°C.

***Annealing.*** Before assembling the origami panel and rigid plate into the metastable module, the panel must be annealed to establish its bistable behavior with the aid of coil springs. As shown in Figure **??**(a), the printed panel is folded inward along its crease lines and temporarily held by a coil spring. This folded panel is annealed in a temperature-controlled oven (EasyComposites OV301) preheated to 110°C for 10 minutes to relieve internal stress in the bent facets. After annealing, the panel can cool to room temperature for 30 minutes to eliminate the residual stress. The horizontal spring is then removed.

In the final step, the head and tail of the coil spring or shape memory alloy (SMA) coil are inserted into the two 3D-printed housings on the origami panel. This setup enables the creation of a bistable structure with two distinct states: a flexible state ([0]) and a stiff state ([1]), as introduced in the main text.

## Note S2: NARMA Computing Setup and Readout Training.

***More Details About NARMA.*** In Task (I), the meta-stable modular structure is utilized as a physical reservoir (adaptive kernel) to explore the optimal input and physical configurations for optimal computing performance. Its computational capability is evaluated through the **Normalized Auto-Regressive Moving Average (NARMA)** system emulation task. NARMA is a widely adopted benchmark for small-scale reservoir systems, as the difficulty of the task is well-characterized by the parameter $N$, which serves as a proxy for the system's memory capacity (2). The NARMA-$N$ system can be expressed by the following equations.

For $N = 2$ (NARMA2) $\hat{y}^2$ could be expressed as :

$$\hat{y}^2(t+1) = 0.4\hat{y}^2(t) + 0.4 \cdot \hat{y}^2(t) \cdot \hat{y}^2(t-1) + 0.6u(t)^3 + 0.1 \quad [1]$$

For $N > 2$ (the general form of NARMA-$N$) $\hat{y}^N$ could be expressed as:

$$\hat{y}^N(t+1) = \alpha\hat{y}^N(t) + 5\beta\hat{y}^N(t)\sum_{j=0}^{N-1} u(t-j) + \gamma u(t-N+1)u(t) + \delta \quad [2]$$

where $u(t)$ is the input signal provided to the system, and $\hat{y}^N(t)$ is the target output for the NARMA-$N$ task. The parameters $\alpha$, $\beta$, $\gamma$, and $\delta$ are constants, whose values must be carefully selected to ensure the stability and boundedness of the system over time. In this study, we adopt the parameters used in Fujii and Nakajima (3), and the physical constraints inherent in the modular structure further help ensure task convergence.

Notably, successful emulation of the NARMA-$N$ system implies that the reservoir possesses a memory span of at least $N$, since the nonlinearity introduced by the second and third terms requires access to the input history over $N$ time steps.

***Procedures of Readouts Training.*** The experimental setup includes a laptop (Dell XPS 13 9340) for generating input commands, a National Instruments DAQ system (NI 9234, National Instruments, Corp.) with a power amplifier, and a large-stroke vibration exciter (APS 113, APS Dynamics, Inc.) that receives the amplified signals. A high-resolution camera (Sony a7C II) captures the displacements of all nodes/markers along the arm. The modular reservoir is connected vibration exciter, with its top end plate fixed to a rigid bar connecting to the exciter as shown in Figure S2. In this way, the modular physical computer could receive base excitation $u(t)$ expressed as a product of three sinusoidal functions where $(f_1, f_2, f_3) = (2.11, 3.73, 4.33)$ Hz times the scaling factor. These three input frequencies were selected to ensure rich temporal excitation while avoiding harmonic overlap. This type of frequency selection was commonly used in previous studies to evaluate a reservoir's computational ability. The nonlinear combination of these incommensurate frequencies creates a broadband input with diverse temporal features, which induces complex dynamics within the system — essential for effective reservoir computing.

Small red markers are attached to the vertices of the origami panel to track the structural dynamics. The camera records their motion at 60 frames per second with 1080p resolution. The recorded footage is post-processed in MATLAB to extract vertical displacements of all nodes as shown in Figure S2. These displacement trajectories form the reservoir state vectors $\mathbf{S}(t) = [s_1(t), s_2(t), \ldots, s_n(t)]^\intercal$, which are then used for the NARMA emulation tasks. Desired outputs $\hat{y}^N(t)$ are obtained by training linear output weights $\mathbf{w}_{\text{out}}^N = [w_0^N, w_1^N, \ldots, w_7^N]^\intercal$ through linear regression such that

$$\mathbf{w}_{\text{out}}^N = [\mathbf{I}\ \mathbf{S}(t)]^+ y^N(t) = \bar{\mathbf{\Phi}}(t)^+ y^N(t), \quad [3]$$

Where $\mathbf{I}$ is a column of ones for calculating the bias term $w_0$, $[\cdot]^+$ is the Moore–Penrose pseudo-inverse to accommodate non-square matrices, and $\hat{\mathbf{y}}^N(t)$ is the target output, which is defined according to the task. Once the readout weights are obtained from training, the reservoir's *predictions* are:

$$\mathbf{O}^{(\mathrm{I})}(t) = w_0 + \sum_{i=1}^{n} w_i^N s_i(t). \quad [4]$$

Figure S3 shows an example of training the adaptive kernel at configuration $\mathbf{C}_5$ to emulate NARMA2 and NARMA10 in parallel, when the input scale is set at the large $\mathbf{A}_3$. After the nodal displacements (reservoir state vectors) are extracted from the video, Equation 3 is applied using $\hat{y}^2(t)$ and $\hat{y}^{10}(t)$ to obtain two groups of readout weights $\mathbf{w}_{\text{out}}^2 = [w_0^2, w_1^2, \ldots, w_n^2]^\intercal$ and $\mathbf{w}_{\text{out}}^{10} = [w_0^{10}, w_1^{10}, \ldots, w_n^{10}]^\intercal$. The orange line in Figure S3 is the target function, and the red line is the reservoir output with trained readout weights. To explicitly show the contribution of body dynamics to the computing, we also compared the computing performance with a simple linear regression layer applied directly to the base input, $\hat{y}^N(t) = w_0^N + w_1^N s_1(t)$, where $w_0^N$ and $w_1^N$ are trained only with the base node displacement from the same data set. The result of this linear regression is shown by the gray dashed line in Figure S3. All computing results with the physical body outperform those with only the linear regression layer, proving the non-negligible contribution of body dynamics to the temporal integration and nonlinearity required by the NARMA tasks. **Besides, to confirms the nonlinear transformation capabilities of our system. Figure S4 represents full spectra from all 15 physical settings (5 configurations $\times$ 3 amplitudes). Additional peaks appear alone with the increase of the input magnitude and the number of module.**

***More Details on MSE.*** Experiments are conducted for each of the five structural configurations under three different input magnitudes, with each setup repeated five times, resulting in a total of 75 trials (5(configurations) $\times$ 3(input magnitude) $\times$ 5(repetitions)). For each trial, the first 600 time steps (10 seconds) are used as a washout phase to allow transient dynamics to settle, followed by 300 time steps (5 seconds) for training, and the final 300 time steps (5 seconds) for evaluation. The errors reported in Figure 4 in the main text represent the average NMSE across all repetitions.

To quantitatively evaluate the predictive performance of the reservoir computing system, the **Normalized Mean Square Error (NMSE)** is used. NMSE measures the deviation of the predicted output from the target values, normalized by the variance of the target signal. It is defined as:

$$\text{NMSE} = \frac{\sum_{i=1}^{N}(y_i - \hat{y}_i)^2}{\sum_{i=1}^{N}(y_i - \bar{y})^2} \quad [5]$$

where $y_i$ denotes the target values, $\hat{y}_i$ represents the predicted values from the reservoir computing model, $\bar{y}$ is the mean of the target signal, and $N$ is the total number of evaluation data points.

***More details on PSI.*** Additionally, to bridge computational performance with the mechanical and frequency domains, the **Peak Similarity Index (PSI)** is introduced. PSI quantifies the discrepancy between the target and predicted signals in the frequency domain by specifically comparing the similarity of their dominant harmonic peaks. The PSI is computed in two steps. First, the magnitudes of the eight dominant harmonic peaks ($f_1$ to $f_8$) are extracted from the frequency spectrum of the target signal and recorded as $A_{f_1}^{\text{target}}$ to $A_{f_8}^{\text{target}}$, shown as the red line in Figure S5. Next, the magnitudes at these same frequencies are extracted from the frequency spectrum of the reservoir outputs, denoted as $A_{f_1}^{\text{predict}}$ to $A_{f_8}^{\text{predict}}$, shown in orange in Figure S5.

The PSI is then calculated as:

$$\text{PSI} = \sum_{i=1}^{8} \frac{A_{f_i}^{\text{predict}}}{A_{f_i}^{\text{target}}}, \quad [6]$$

where $\frac{A_{f_i}^{\text{predict}}}{A_{f_i}^{\text{target}}}$ represents the similarity at the $i$th dominant frequency $f_i$. A PSI value close to 8 indicates high spectral similarity between the targeted signals and reservoir output, while a value near 0 suggests the reservoir fails to capture the targeted frequency characteristics.

Figure S6 summarize the $\frac{A_{f_i}^{\text{predict}}}{A_{f_i}^{\text{target}}}$ values at the first 8 dominant harmonic peaks corresponding to the five different configurations for the NARMA2, NARMA5, NARMA10, NARMA15, and NARMA20 tasks. This visualization illustrates how PSI is computed across different emulation tasks and reservoir configurations. By directly evaluating the preservation of dominant frequency content, PSI offers an interpretable metric for assessing the ability of the physical reservoir to capture essential dynamical features, thereby linking computational accuracy with the underlying mechanical configuration.

***Correlation between PSI and NMSE.*** Figure S7 shows a correlation analysis between PSI and NMSE across all five NARMA tasks (NARMA 2, 5, 10, 15, and 20). Each subplot displays the PSI–NMSE relationship for one task using 15 different physical configurations. The results consistently show a strong negative relationship between PSI and NMSE for all tasks, indicating that higher spectral similarity (as measured by PSI) correlates with better emulation performance (meaning lower NMSE). The correlation coefficients range from –0.71 to –0.87, all statistically significant with $p<0.01$. These findings support the usefulness of PSI as a reliable indicator of task performance across different physical reservoirs.

**Note S3: Comparing Adaptive Reservoir to Prior Works.** We compare the NARMA performance of our adaptive kernel with that of a soft silicone arm reported in (4), which has been shown to exhibit strong computational performance, even outperforming computer-simulated reservoirs in certain scenarios. In that study, the authors evaluated the NARMA tasks of order 2, 5, 10, 15, and 20 under various time scaling parameters, which modulate the phase velocity of the input time series. The minimum usable scaling factor in their experiments was 100, constrained by motor overheating at lower values.

In contrast, our system utilizes a vibration shaker for actuation, allowing for much higher input frequencies. However, our temporal resolution is constrained by the camera sampling frequency at 60 Hz. Therefore, we compare our NARMA emulation results—based on the optimal reservoir configuration—with those from (4) obtained at a time scaling of 100.

The normalized mean square errors (NMSE) for NARMA tasks of orders 2, 5, 10, 15, and 20, computed using our adaptive kernel at the optimal configuration, the soft silicone body, and the artificial echo state network (ESN) reservoir in the reference study, are summarized in Table S1.

| Task | $\mathrm{NMSE}^{\mathrm{min}}_{\mathrm{Modular}}$ | $\mathrm{NMSE}^{\mathrm{system}}_{\mathrm{Reference}}$ | $\mathrm{NMSE}^{\mathrm{ESN}}_{\mathrm{Reference}}$ |
|---|---|---|---|
| NARMA2 | $1.73 \pm 0.05$ $(\times 10^{-5})$ | $1.36 \pm 0.07$ $(\times 10^{-5})$ | $0.94 \pm 0.20$ $(\times 10^{-5})$ |
| NARMA5 | $0.69 \pm 0.04$ $(\times 10^{-3})$ | $1.50 \pm 0.04$ $(\times 10^{-3})$ | $1.72 \pm 0.90$ $(\times 10^{-3})$ |
| NARMA10 | $1.49 \pm 0.11$ $(\times 10^{-3})$ | $1.97 \pm 0.02$ $(\times 10^{-3})$ | $1.75 \pm 0.31$ $(\times 10^{-3})$ |
| NARMA15 | $3.83 \pm 0.07$ $(\times 10^{-3})$ | $2.81 \pm 0.03$ $(\times 10^{-3})$ | $2.87 \pm 0.57$ $(\times 10^{-3})$ |
| NARMA20 | $1.07 \pm 0.02$ $(\times 10^{-3})$ | $1.66 \pm 0.01$ $(\times 10^{-3})$ | $1.51 \pm 0.12$ $(\times 10^{-3})$ |

**Table S1. Comparison of NMSE for different NARMA tasks using the optimized adaptive kernel, soft silicone reservoir, and artificial reservoir network from (4).**

Surprisingly, although our adaptive kernel is neither as soft as the silicone arm, nor specifically designed for emulation tasks like the artificial reservoir, its performance is comparable to or even exceeds the reference systems in NARMA5, NARMA10, and NARMA20 tasks. This result highlights that the ability to realize physical computing is not limited to specially designed soft structures but can also emerge from generic, reconfigurable, and mechanically adaptable platforms. While soft materials generally show material nonlinearity that is naturally advantageous for reservoir computing, our origami reservoir — though made from relatively stiff materials — demonstrates strong geometric nonlinearity caused by features like bistability and finite rotations of origami folds and creases. This supports a broader perspective that the effectiveness of a mechanical reservoir depends not solely on stiffness or softness but on its capacity to produce diverse, high-dimensional, and nonlinear dynamic responses. Taking one step further, our findings indicate that what matters most is the characteristics of the resulting dynamic behavior — especially in terms of frequency content and state space correlations, as measured by the spectral and spatial metrics introduced in our work. These metrics can serve as more reliable indicators of computational richness than stiffness alone, and we believe this view offers a valuable design consideration for a wide range of physical reservoirs.

**Note S4: Additional Study by Configuring a 5-segment Adaptive Kernel.** We demonstrate how to configure the adaptive kernel to maximize its computing performance through five structural configurations in the *Task (I)* section, involving a different number of modules. Interestingly, even when the number of modules is fixed, the computing capability still varies depending on the specific combination of flexible and stiff states, as shown in Figure S6. In this set of additional experiments, both the experimental setup and the training procedure follow the same protocol described in the *Task I* section.

Specifically, we fix the number of modules to five and define six distinct configurations by varying the arrangement of flexible and stiff modules. Each configuration is subjected to input signals under three different magnitude conditions, resulting in a total of $6 \times 3 = 18$ experimental setups for the NARMA-$N$ emulation tasks.

Figure S8 summarizes the NMSE results from all 18 experimental setups across five NARMA tasks (NARMA2, 5, 10, 15, and 20). The orange bars represent the averaged NMSE for each configuration, while the overlaid line plots indicate the corresponding Peak Similarity Index (PSI). Consistent with the findings in Task (I) section in the main text, computing performance generally improves (i.e., NMSE decreases) with increased input magnitude. Furthermore, a positive correlation is observed between PSI and computational accuracy, further supporting the use of PSI as a spectral-domain performance indicator.

Finally, Figure S10 provides a comprehensive overview of the optimal configuration for NARMA emulation tasks ranging from order 2 to 20 under different input magnitudes. When the input magnitude is small, configurations with greater flexibility (i.e., the softest configuration) tend to give better computational output. In contrast, when the input magnitude increases, the optimal configuration varies across the 19 different NARMA tasks. Notably, all six configurations can serve as the optimal structure for some target functions, highlighting the adaptability and task-specific tunability of the adaptive modular reservoir.

## Note S5: Readout Training for Payload Mass Estimation.

***Experiments Setup.*** In Task (II), the experimental setup remains the same as in Task (I), as shown in Figure S18. The adaptive kernel is connected to a shaker that provides base excitation. The only modification is that an additional payload mass (0 g, 50 g, 90 g, 130 g, or 170 g) is attached to the last module. Consequently, the structural dynamics of the system are altered depending on the attached mass. The adaptive kernel is expected to estimate the payload mass based on its dynamic responses (i.e., through the displacements of all nodal markers). Besides,five input frequencies ranging from 2 Hz to 10 Hz were used. The lower bound of 2 Hz was selected because, at this frequency, the system shows minimal elastic deformation — resulting in motion dominated by rigid-body dynamics, which is not suitable for computation. The upper bound of 10 Hz was chosen due to experimental limitations: as frequency increases, the input amplitude from the shaker significantly decreases, limiting the system's dynamic response.

Here, we use configuration $\mathbf{C}_5$ as an example to illustrate the process of readout training for mass estimation using different training data sizes. For each payload mass, the excitation test is repeated 10 times—one trial is used for training, and the remaining nine trials are reserved for testing. The vertical displacements of 40 nodes are extracted from the recorded videos to form the state vectors:

$$\mathbf{S}_{\text{mass}}^{\text{train/test}}(t) = [s_1(t),\ s_2(t),\ \ldots,\ s_{40}(t)]^\intercal, \tag{7}$$

for both training and testing. Thus, we obtain one set of training state vectors for each payload mass:

$$\mathbf{S}_{0g}^{\text{train}},\quad \mathbf{S}_{50g}^{\text{train}},\quad \mathbf{S}_{90g}^{\text{train}},\quad \mathbf{S}_{130g}^{\text{train}},\quad \mathbf{S}_{170g}^{\text{train}}, \tag{8}$$

and nine sets of testing data:

$$\mathbf{S}_{0g}^{\text{test}},\quad \mathbf{S}_{50g}^{\text{test}},\quad \mathbf{S}_{90g}^{\text{test}},\quad \mathbf{S}_{130g}^{\text{test}},\quad \mathbf{S}_{170g}^{\text{test}}. \tag{9}$$

The training efficiency can be improved by reducing the number of payload mass conditions included in the training data. Specifically, Figure S11(c) presents comprehensive results when the arm is trained using data from 1, 2, 3, 4, or all 5 payload masses. Correspondingly, the training size is reduced to 1/5, 2/5, 3/5, 4/5, and 5/5 of the full dataset, respectively.

***Training with One End Mass.*** Figure S11(a) details the results when using only a single training state vector, $\mathbf{S}_{0g}^{\text{train}}$, for readout training.

The target function for this training is set as a constant function equal to the training mass value (0 g), represented by the blue dashed line in the second plot:

$$\hat{y}(t) = 0, \quad \text{for } 0 < t < 5\,\text{s}. \tag{10}$$

A set of readout weights $\mathbf{w}_{\text{out}}^1 = [w_0^1, w_1^1, \ldots, w_{40}^1]^\intercal$ is trained through the linear regression Equation (3) such that:

$$\hat{y}(t) \approx \mathbf{w}_{\text{out}}^1 \mathbf{S}_{0g}^{\text{train}}(t). \tag{11}$$

The second subplot of Figure S11 illustrates the good agreement between the reservoir output (orange curve) and the target function (blue dashed line).

After training, the obtained readout weights $\mathbf{w}_{\text{out}}^1 = [w_0^1, w_1^1, \ldots, w_{40}^1]^\intercal$ are applied to the testing datasets to evaluate estimation performance. The colorful lines in the third subplot represent the testing outputs:

$$y_{0g}^{\text{test}} = \mathbf{w}_{\text{out}}^1 \mathbf{S}_{0g}^{\text{test}},\quad y_{50g}^{\text{test}} = \mathbf{w}_{\text{out}}^1 \mathbf{S}_{50g}^{\text{test}},\quad y_{90g}^{\text{test}} = \mathbf{w}_{\text{out}}^1 \mathbf{S}_{90g}^{\text{test}},\quad y_{130g}^{\text{test}} = \mathbf{w}_{\text{out}}^1 \mathbf{S}_{130g}^{\text{test}},\quad y_{170g}^{\text{test}} = \mathbf{w}_{\text{out}}^1 \mathbf{S}_{170g}^{\text{test}}. \tag{12}$$

The estimated mass value $\bar{y}_{\text{out}}$ is computed by averaging the reservoir outputs over all time steps (5 s) as:

$$\bar{y}_{\text{out}} = \frac{1}{T} \sum_{t=1}^{T} y_{\text{out}}(t). \tag{13}$$

The results show that the estimated outputs for all different payload masses are close to zero (far from the ground truth), demonstrating that training with only one mass condition is insufficient for accurate mass estimation across different payloads.

***Training with Two End Masses.*** We increase the training data by using the nodal displacement from two end masses to improve the reservoir's performance. Specifically, two training state vectors corresponding to the two extreme payload conditions—no payload (0 g) and maximum payload (170 g)—are compiled for training, as shown in Figure S11(b). The training vectors are concatenated in time to form a single sequence:

$$\mathbf{S}^{\text{train}} = \begin{bmatrix} \mathbf{S}_{0\,\text{g}}^{\text{train}} \\ \mathbf{S}_{170\,\text{g}}^{\text{train}} \end{bmatrix}, \tag{14}$$

as depicted in the top row of each subplot in Figure S11. The corresponding target function is defined as:

$$\hat{y}(t) = \begin{cases} 0, & \text{for } 0 < t < 5\,\text{s}, \\ 170, & \text{for } 5 < t < 10\,\text{s}. \end{cases} \quad [15]$$

Notably, the total training duration is extended from 5 s to 10 s. A new set of readout weights $\mathbf{w}_{\text{out}}^2 = [w_0^2, w_1^2, \ldots, w_{40}^2]^\intercal$ is obtained by applying the linear regression Equation (3) to the concatenated training data, resulting in:

$$\hat{y}(t) \approx \mathbf{w}_{\text{out}}^2 \mathbf{S}^{\text{train}}(t). \quad [16]$$

The second subplot in Figure S11 demonstrates the close alignment between the reservoir output (orange curve) and the target function (blue dashed line).

The testing process remains the same: the newly trained readout weights are applied to the testing datasets. The third subplot in Figure S11 shows the reservoir outputs for each testing condition, namely $y_{0g}^{\text{test}}$, $y_{50g}^{\text{test}}$, $y_{90g}^{\text{test}}$, $y_{130g}^{\text{test}}$, and $y_{170g}^{\text{test}}$.

Compared to the case trained with only one mass, the adaptive kernel now demonstrates significantly improved separability between different payloads, with only slight deviations between the target and actual outputs.

***Training with Five End Masses.*** For the most comprehensive training scenario, we use the nodal displacement from all five available payload masses—0 g, 50 g, 90 g, 130 g, and 170 g—for readout training. The corresponding state vectors are concatenated sequentially to form the full training sequence:

$$\mathbf{S}^{\text{train}} = \begin{bmatrix} \mathbf{S}_{0\,\text{g}}^{\text{train}} \\ \mathbf{S}_{50\,\text{g}}^{\text{train}} \\ \mathbf{S}_{90\,\text{g}}^{\text{train}} \\ \mathbf{S}_{130\,\text{g}}^{\text{train}} \\ \mathbf{S}_{170\,\text{g}}^{\text{train}} \end{bmatrix}. \quad [17]$$

The corresponding target function is defined as a piecewise-constant signal matching the payload weight for each time interval:

$$\hat{y}(t) = \begin{cases} 0, & \text{for } 0 < t < 5\,\text{s}, \\ 50, & \text{for } 5 < t < 10\,\text{s}, \\ 90, & \text{for } 10 < t < 15\,\text{s}, \\ 130, & \text{for } 15 < t < 20\,\text{s}, \\ 170, & \text{for } 20 < t < 25\,\text{s}. \end{cases} \quad [18]$$

Thus, the total training duration increases to 25 s. A new set of readout weights $\mathbf{w}_{\text{out}}^5 = [w_0^5, w_1^5, \ldots, w_{40}^5]^\intercal$ is trained using the linear regression Equation (3), resulting in:

$$\hat{y}(t) \approx \mathbf{w}_{\text{out}}^5 \mathbf{S}^{\text{train}}(t). \quad [19]$$

As shown in Figure S11(c), the reservoir output closely follows the target function during training, confirming that the system successfully encodes the payload-dependent dynamics.

The same trained readout weights are then applied to the testing datasets. The third subplot in Figure S11 shows the testing results for all payload conditions. The outputs exhibit clear separability across different masses, with minimal deviations from the true mass values. This result indicates that using a broader range of training conditions enhances the reservoir's generalization ability and improves mass estimation accuracy across unseen payloads.

***Distribution of readout weights.*** The distribution of readout weights varies greatly depending on both the input excitation frequency and the amount of training data. There are no fixed nodes that consistently dominate the readout in all situations, suggesting that different physical configurations activate different parts of the reservoir to support computation.

However, one general trend was observed. For the softer structure (e.g., Configuration 5), we often see larger readout weights concentrated near the top and bottom nodes, indicating that fewer nodes are sufficient to extract the necessary information for the payload classification task. In contrast, the stiffer structure (Configuration 6) tends to distribute the readout weights more evenly across the nodes, suggesting that broader spatial integration of information is needed. This behavior is shown in Supplementary Figure S12, which displays the normalized readout weight distributions for C5 and C6 under 10 Hz excitation with all payload classes included in training.

***Definition of spatial correlation.*** The displacement data from 40 nodes distributed along the adaptive kernel's body are recorded to characterize its dynamic properties. To quantitatively describe the relationships between these nodes, a correlation matrix $\mathbf{C}_{\text{orr}}$ is constructed, where each element $C_{\text{orr},ij}$ represents the correlation coefficient between the displacement time series of node $i$ and node $j$. The correlation matrix is formally defined as:

$$\mathbf{C}_{\text{orr}} = \begin{bmatrix} C_{\text{orr},11} & C_{\text{orr},12} & \dots & C_{\text{orr},1N} \\ C_{\text{orr},21} & C_{\text{orr},22} & \dots & C_{\text{orr},2N} \\ \vdots & \vdots & \ddots & \vdots \\ C_{\text{orr},N1} & C_{\text{orr},N2} & \dots & C_{\text{orr},NN} \end{bmatrix} \quad [20]$$

where $N = 40$, and each entry $C_{\text{orr},ij}$ is calculated as:

$$C_{\text{orr},ij} = \frac{\text{cov}(s_i, s_j)}{\sigma_{s_i}\sigma_{s_j}}. \quad [21]$$

Here, $s_i$ and $s_j$ denote the displacement data from nodes $i$ and $j$, respectively, $\text{cov}(s_i, s_j)$ is their covariance, and $\sigma_{s_i}$, $\sigma_{s_j}$ are the standard deviations.

The correlation index $R_i$ for each node $i$ is then defined as the sum of correlations between node $i$ and all other nodes in the structure, given by:

$$R_i = \sum_{j=1}^{N} C_{\text{orr},ij}. \quad [22]$$

The average correlation index of the whole structure, Avg. CI, is then defined as the average of the correlation index for all nodes.

$$Avg.CI = \frac{\sum_{i=1}^{N} R_i}{N} \quad [23]$$

The correlation index provides an insightful measure indicating each node's average connectivity or interaction strength with the overall structural dynamics, thus enabling the assessment of nodes with significant influence or sensitivity within the structure.

## Note S6: Setup and Readout Training for the Adaptive Robotic Kernel.

***More details about the SMA actuation setup, mechatronic diagrams.*** The robotic kernel designed for manipulation tasks features a four-module setup, as introduced in Section Task (III). The Shape Memory Alloys (SMAs) used in this task are Nitinol helical springs (Kellogg's Research Labs, wire diameter: 0.5mm, mandrel diameter: 4.75mm, transition temperature: 45°C). The thermo-mechanical behavior of SMA—including response hysteresis, temperature-dependent actuation delay, and strong coupling between thermal and mechanical domains—has indeed been studied extensively and is also documented in our prior work(5). Each spring is cut into twelve segments, resulting in SMA coils of 20mm in length, which are then integrated into the modular robotic system to enable thermally-induced actuation. Each Shape Memory Alloy (SMA) embedded in these modules is labeled as $S_{i-j}$, where $i$ (with values 1, 2, 3, or 4) indicates the module number, and $j$ (with values 1, 2, or 3) denotes the column of the SMA. In total, there are four SMAs arranged in each column, which are connected in series. This setup includes three arrays of SMAs, as depicted in Figure S14.

Each SMA array is responsible for inducing motion in one bending direction of the arm. These arrays are activated sequentially using a PWM signal generated by an Arduino Uno. The mechatronics diagram controlling the robotic arm is illustrated in Figure S14(a). The arm operates on a control logic that cycles through each SMA array, with an activation period of 0.5 seconds and a total cycle time of 1.5 seconds. The Arduino outputs PWM signals to three channels linked to one SMA through an MDD10A dual-channel motor driver. Since the MDD10A only supports two channels, two DC motors drivers are included in the setup. A 6V power supply, specifically the BK Precision 9131C rated at 5A, provides ample current to heat the SMAs to their transformation temperature quickly. Limited by the physical properties of the SMA actuators, Only two actuation frequency are chosen in this task. If the input frequency is too low, the SMA wires do not heat enough to cause effective bending. Conversely, at high frequencies, the wires risk overheating and damage. Based on this trade-off and repeated trial-and-error, we chose 0.33 Hz and 0.67 Hz as the operating frequencies to safely and effectively activate the SMA-based system

During each actuation cycle, SMA1 is activated first, followed by SMA2 and SMA3, allowing the robotic arm to bend sequentially at a high frequency without the risk of overheating. This configuration enables the robotic system to exhibit dynamic and cyclic motion patterns with minimal mechanical complexity, effectively utilizing the intrinsic dynamics of the whole structure for input command reconstruction and payload information identification.

***Readout Training For SMA Input Reconstruction.*** We attach four items to the robotic kernel's free end, one at a time, to train the readouts for the input reconstruction task (Figure S16). After the SMAs were heated to their transition temperature, the robotic arm initially exhibited large deformations toward the left-front and right-front directions during the transient phase. It then swung slightly around a balanced bending position oriented toward the front. This swinging motion was induced by the alternating actuation of SMA arrays 1, 2, and 3. The dynamic responses, represented by red marker's displacement distributed along the kernel, were captured by a camera and used to construct the state vectors $\mathbf{S}(t) = [s_1(t), s_2(t), \ldots, s_{40}(t)]^\intercal$ for readout training.

The three input PWM signals generated by the Arduino, shown in gray in Figure S15, were used as target functions $y$:

$$y_i(t) = \text{Input}_i \quad (i = 1, 2, 3), \tag{24}$$

and three distinct sets of readout weights, $\mathbf{w}^1_{\text{out}}$, $\mathbf{w}^2_{\text{out}}$, and $\mathbf{w}^3_{\text{out}}$, were trained in parallel using Equation (3). The reservoir outputs during the training phase are shown in blue in Figure S15, illustrating the system's ability to rebuild the target inputs.

To evaluate the reconstruction performance, we computed the Mean Squared Error (MSE) between the predicted signals and the ground truth inputs during the testing phase:

$$\text{MSE}_i = \frac{1}{T}\sum_{t=1}^{T}\left(\hat{y}^i_3(t) - y^i_3(t)\right)^2, \quad i = 1, 2, 3. \tag{25}$$

In each experimental condition (i.e., each payload setup), the robotic kernel was actuated for 30 seconds, and the experiment was repeated 10 times. For each trial, the first 15 seconds were used for training, and the remaining 15 seconds were used for testing. The Mean Squared Error (MSE) was calculated over the testing interval of each trial. In the bar plots shown in Figure S16, the average MSE across the 10 trials is reported for each condition, and the error bars indicate the minimum and maximum MSE values observed. This evaluation protocol captures the variability of the system under repeated actuation and demonstrates the robustness of its reconstruction capability across different dynamic regimes and load conditions.

Overall, the results suggest that higher-frequency actuation ($\mathbf{\Omega}_7$) is more beneficial for proprioceptive information estimation. This could be attributed to the fact that, under higher-frequency stimulation, the robotic kernel's vibration becomes more synchronized with the input command, thereby enabling more accurate regeneration of the original control signal. Furthermore, it is observed that the reconstruction accuracy is notably higher for input commands applied to columns 1 and 3 when the arm is in configuration $\mathbf{C}_8$. This is likely because column 2 remains stiff (state '1') and cannot move, introducing asymmetry in the actuation pattern. However, this immobility stabilizes the overall bending behavior, contributing to consistent dynamic responses that generally make $\mathbf{C}_8$ outperform $\mathbf{C}_7$.

***Readout Training for Payload Weight and Orientation Classification.*** For the payload perception task using the SMA-actuated robotic kernel, we use different workshop tools, such as pliers, screwdrivers, and hammers, as perception targets to bring the task closer to practical applications. The objective is for the reservoir-enabled robotic arm to not only classify the object being held but also infer its orientations—for instance, determining whether a hammer is oriented with its head towards the front, left, or right. This form of exteroception is achieved through a two-step learning procedure: (1) payload weight estimation for coarse classification and (2) orientation classification for finer-grained recognition. The arm can infer both the object type and its spatial configuration because its dynamic response is influenced by the payload's weight and the centroid location relative to the grasping point.

The arm is trained to perform payload weight estimation in the first step, following the method in the previous Task (II). For each of the six payload scenarios shown in Figure S13, 5 seconds of displacement data from all red markers along the arm are recorded. These sequences are concatenated to form a single state vector:

$$\mathbf{S}(t) = \begin{bmatrix} \mathbf{S}^{\text{item}_1}(t) \\ \mathbf{S}^{\text{item}_2}(t) \\ \mathbf{S}^{\text{item}_3}(t) \\ \mathbf{S}^{\text{item}_{4L}}(t) \\ \mathbf{S}^{\text{item}_{4R}}(t) \\ \mathbf{S}^{\text{item}_{4F}}(t) \end{bmatrix}, \qquad [26]$$

as illustrated in Figure S17(a). The corresponding target function is defined to represent the estimated effective weight of each payload:

$$\hat{\mathbf{y}}_4(t) = \begin{cases} 61.90, & \text{for } 0 < t < 5\,\text{s}, \\ 100.64, & \text{for } 5 < t < 10\,\text{s}, \\ 213.95, & \text{for } 10 < t < 15\,\text{s}, \\ 161.25, & \text{for } 15 < t < 20\,\text{s}, \\ 161.25, & \text{for } 20 < t < 25\,\text{s}, \\ 161.25, & \text{for } 25 < t < 30\,\text{s}. \end{cases} \qquad [27]$$

The readout weights $\mathbf{w}^6_{\text{out}}$ are trained using these input–output pairs. As shown in Figure S17(b), the reservoir output aligns well with the target function, demonstrating effective encoding of weight-based classification.

After training, each payload condition was tested in 10 repeated trials, each lasting 30 seconds. This results in $6 \times 10$ groups of state vectors. During testing, the reservoir outputs were computed using the pre-trained readout weights via $y_4(t) = \mathbf{w}^6_{\text{out}}\mathbf{S}_{\text{test}}(t)$. The Mean Squared Error (MSE) was computed over each testing interval. As shown in Figure S18(a) and (b), the average MSE across all trials remains below 5% for both robotic configurations $\mathbf{C}_7$ and $\mathbf{C}_8$. Notably, lower actuation frequency (0.33 Hz) resulted in significantly smaller variance.

Building on the weight estimation task results, we further train the robotic arm kernel to infer the hammer's orientation, provided that the average predicted weight lies between 140 g and 180 g—indicating that item 4 (the hammer) is being held. We extract displacement data corresponding to the three orientations (left, front, right) of the hammer, as shown in Figure S13. The sequences are concatenated to form the orientation-specific training input as shown in Figure S17(d):

$$\mathbf{S}(t) = \begin{bmatrix} \mathbf{S}^{\text{item}_{4L}}(t) \\ \mathbf{S}^{\text{item}_{4F}}(t) \\ \mathbf{S}^{\text{item}_{4R}}(t) \end{bmatrix}. \qquad [28]$$

This task is treated as a classification problem with three discrete direction labels. The target function for direction perception is defined as:

$$\hat{\mathbf{y}}_5(t) = \begin{cases} -1, & \text{if the hammer is heading left,} \\ 0, & \text{if the hammer is heading front,} \\ 1, & \text{if the hammer is heading right.} \end{cases} \qquad [29]$$

The readout weights $\mathbf{w}^7_{\text{out}}$ are trained using these labels and corresponding state vectors. The training results shown in Figure S17(e) confirm that the reservoir successfully captures directional information encoded in the dynamic response.

Testing is conducted over 10 repeated trials for each hammer orientation. The MSE of classification results is reported in Figure S18(c) and (d). Across both configurations, the system reliably distinguishes between different orientations with low variance, particularly under configuration $\mathbf{C}_7$. Additionally, slower actuation stabilizes the dynamic profile and reduces prediction variance. This implies that lower-frequency motion leads to richer, more separable dynamics for exteroceptive tasks such as orientation inference.

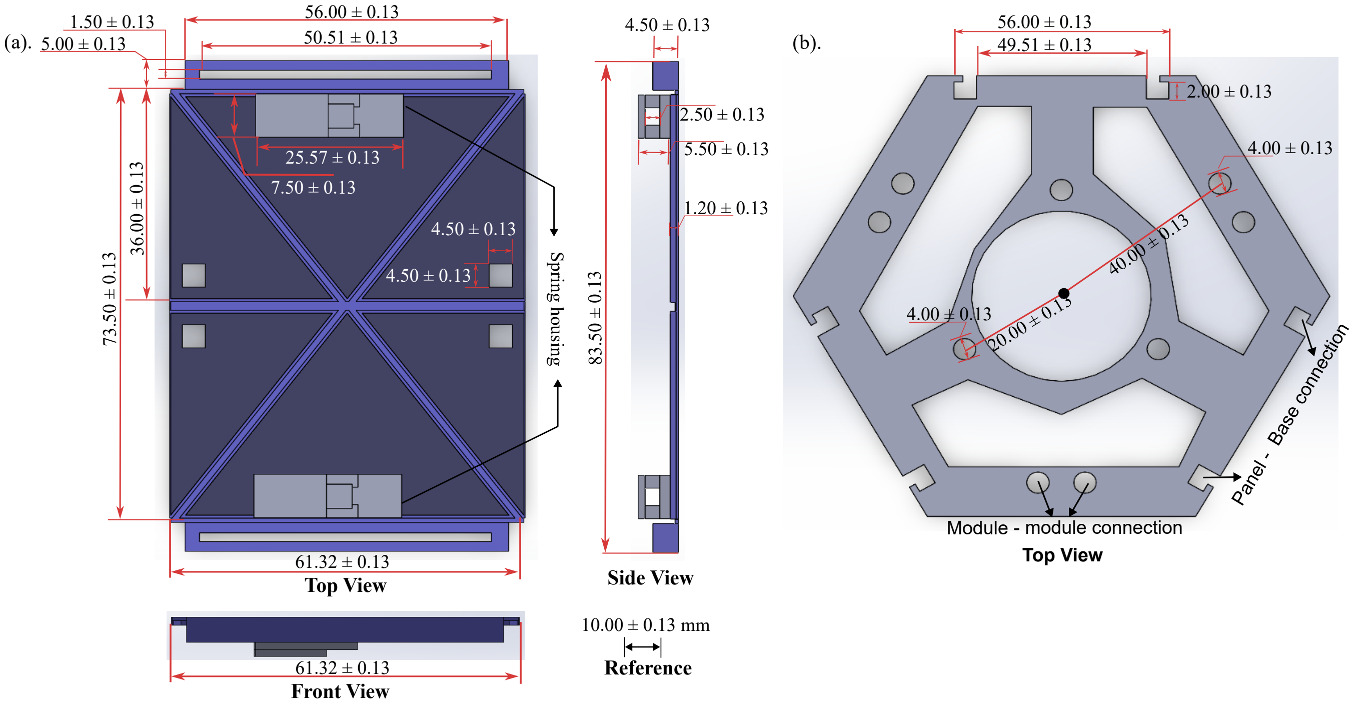


**Fig. S1.** CAD drawings of the origami panel and base plate. (a) Dimensions of the origami panel in top, side, and front views. (b) Dimensions of the rigid base plate used for assembling three panels into a single module and connecting multiple modules.

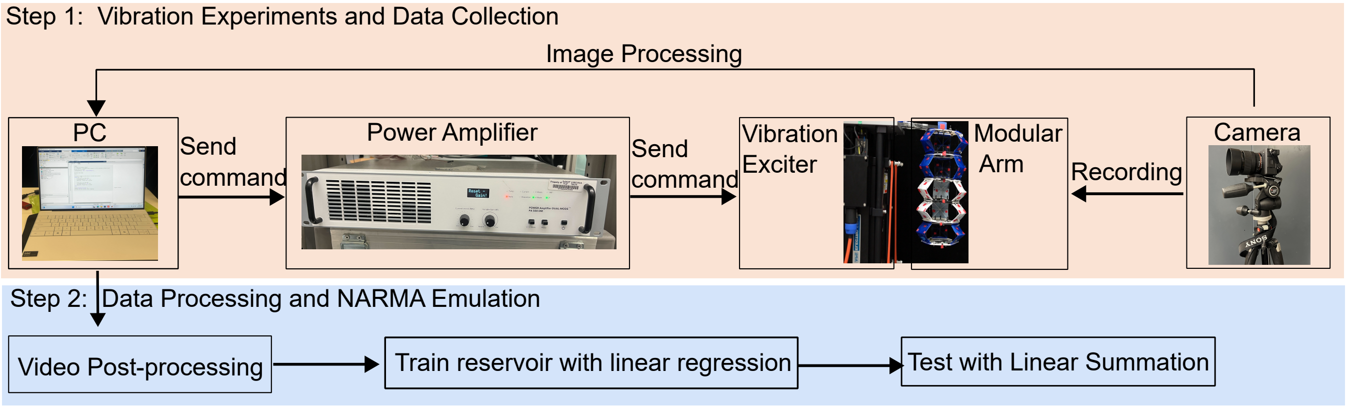


**Fig. S2.** Work flow chart of the vibration experiments for tasks (I) and (II)

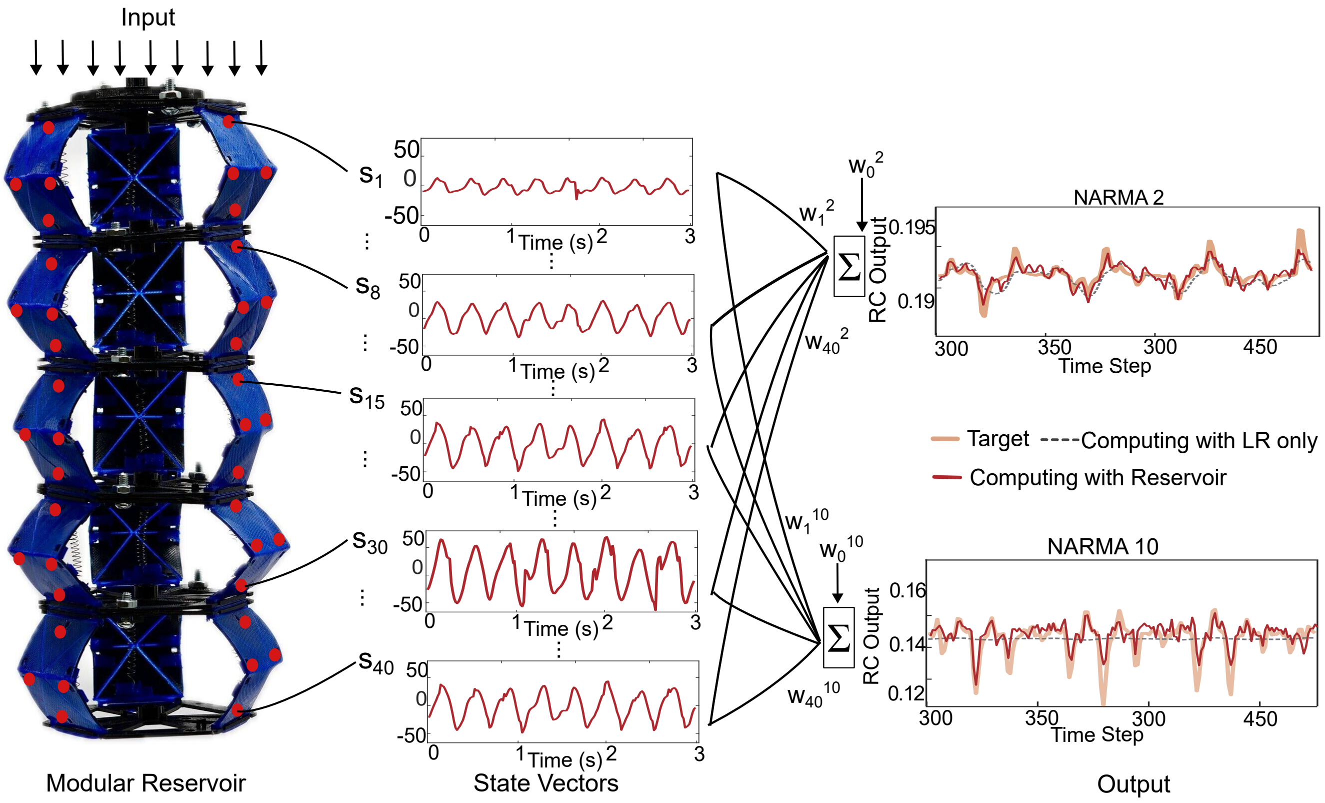


**Fig. S3.** Example of training the adaptive kernel at $\mathbf{C}_5$ to emulate NARMAR2 and NARMA10 in parallel. The modular structure will vibrate with the shaker after receiving an input signal from the computer. The camera records the displacement of all red markers attached to the structure. Then, readout weights for NARMA2 and NARMA10 are trained in parallel using linear regression.

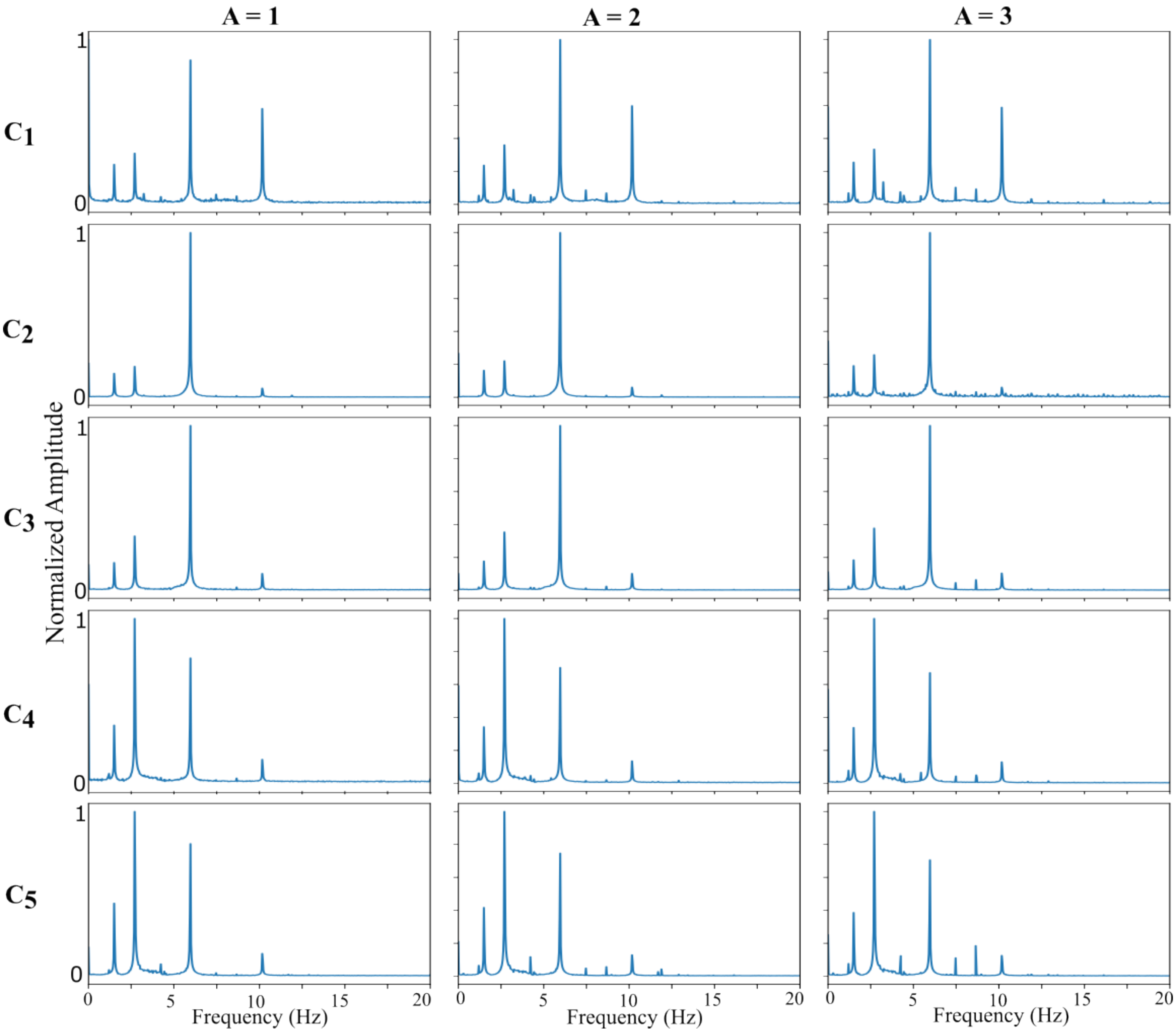


**Fig. S4.** Example of training the adaptive kernel at $\mathbf{C}_5$ to emulate NARMAR2 and NARMA10 in parallel. The modular structure will vibrate with the shaker after receiving an input signal from the computer. The camera records the displacement of all red markers attached to the structure. Then, readout weights for NARMA2 and NARMA10 are trained in parallel using linear regression.

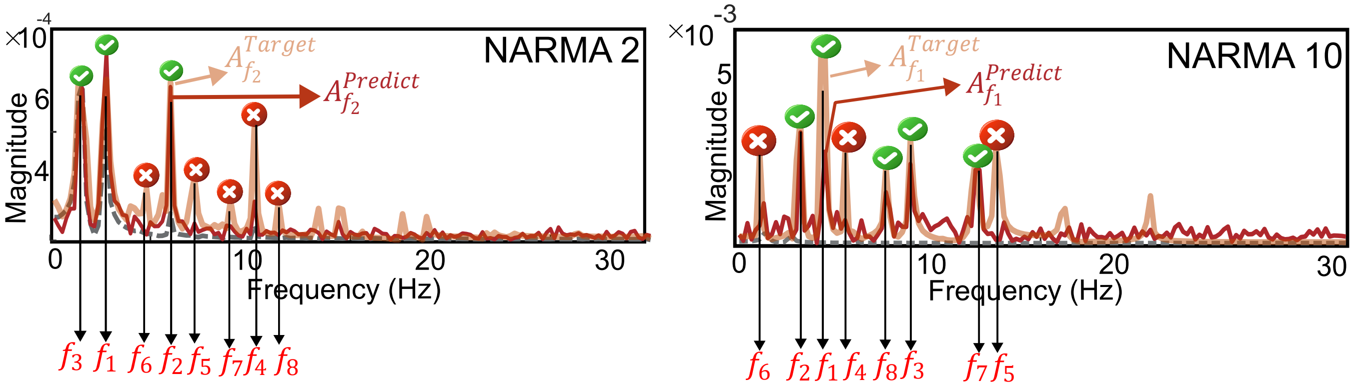


**Fig. S5.** Spectral analysis corresponding to the emulation results of NARMA2 and NARMA10 shown in Figure S3.

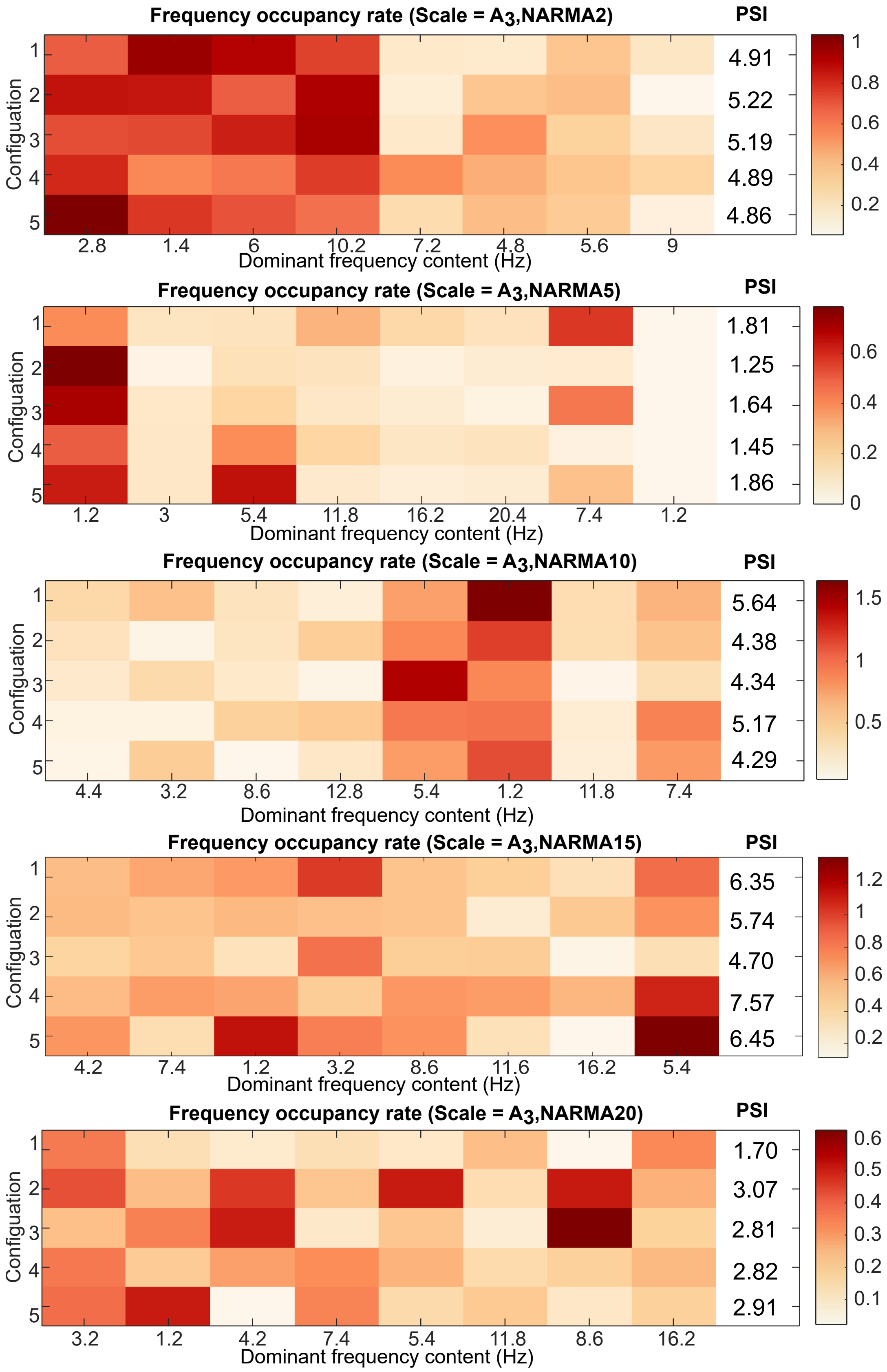


**Fig. S6.** Frequency occupancy rates ($\frac{A_{f_i}^{\text{predict}}}{A_{f_i}^{\text{target}}}$) at the first eight dominant harmonic peaks of NARMA2, NARMA5, NARMA10, NARMA15, and NARMA20 tasks, evaluated using five different physical configurations. Each row in the matrix corresponds to a distinct reservoir configuration. The last column in each matrix displays the PSI value for the respective configuration, representing the overall spectral similarity between the predicted and target signals after completing the emulation task. This figure demonstrates an example of how PSI is derived, corresponding to the summary presented in Figure ??(b).

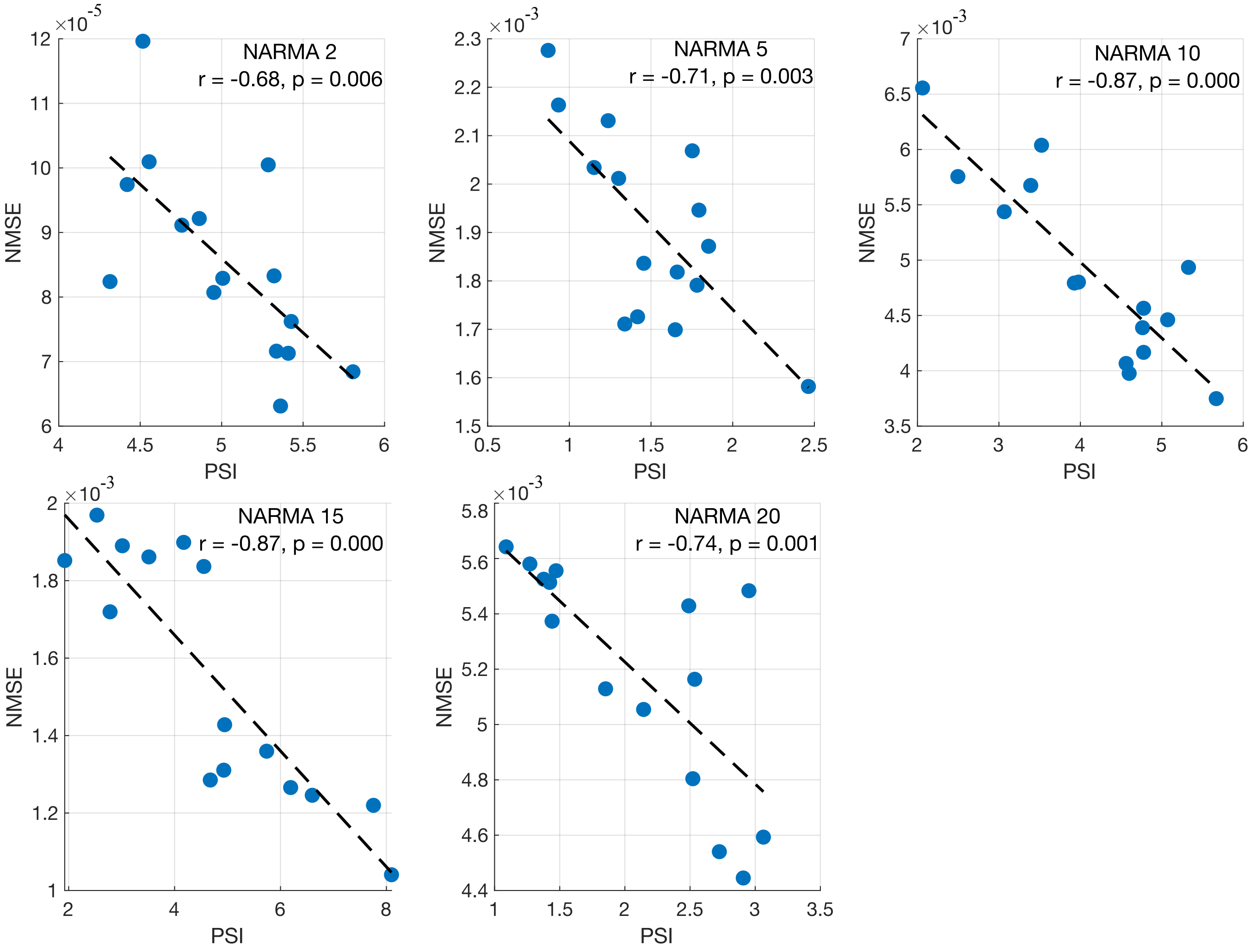


**Fig. S7.** Correlation between PSI and NMSE for all 15 physical configurations across 5 NARMA tasks.

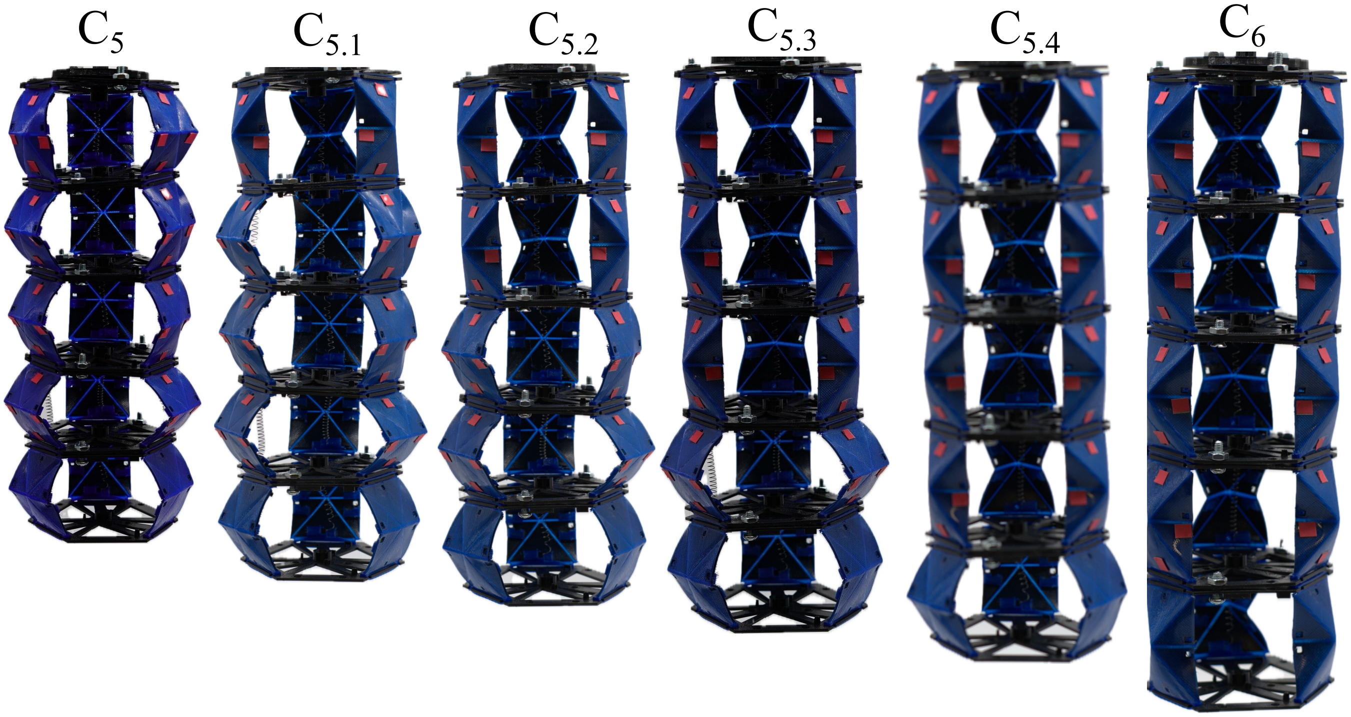


**Fig. S8.** Six different configurations of the five-module kernel for adaptive computing. Each configuration represents a unique arrangement of flexible and stiff modules.

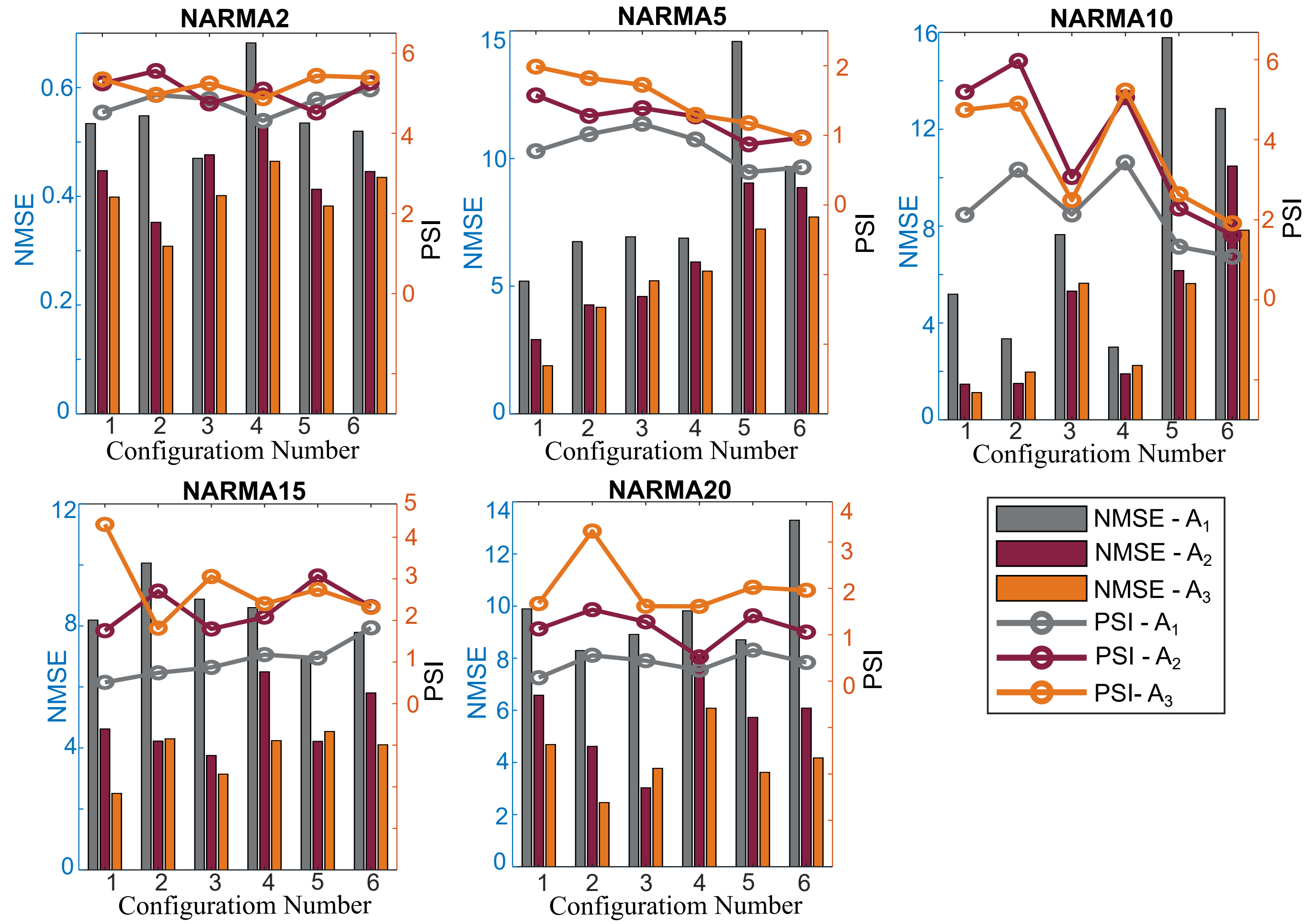


**Fig. S9.** Averaged NMSE (bar plot) and corresponding PSI (line plot) for NARMA tasks of order 2, 5, 10, 15, and 20 across six different five-module configurations and three input magnitudes.

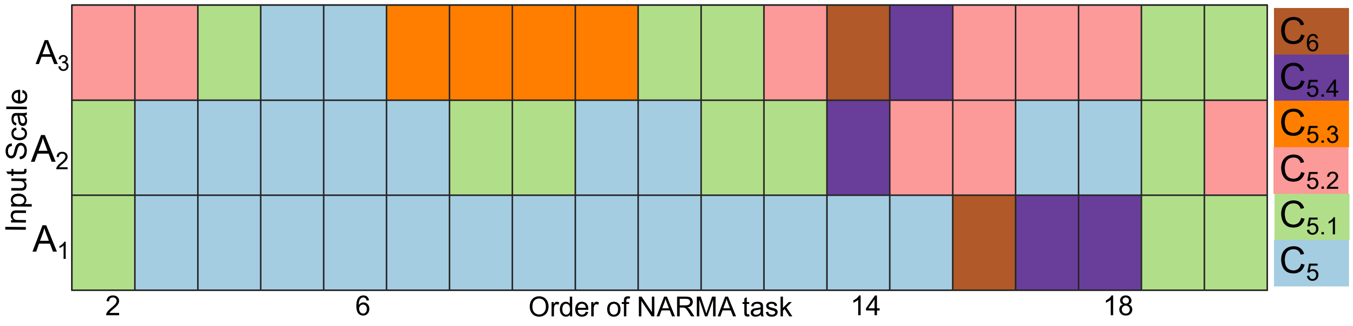


**Fig. S10.** Optimal configuration matrix for NARMA emulation tasks of order 2 to 20 across different input magnitudes. Each cell indicates the best-performing configuration among the six tested for a given task and input scale.

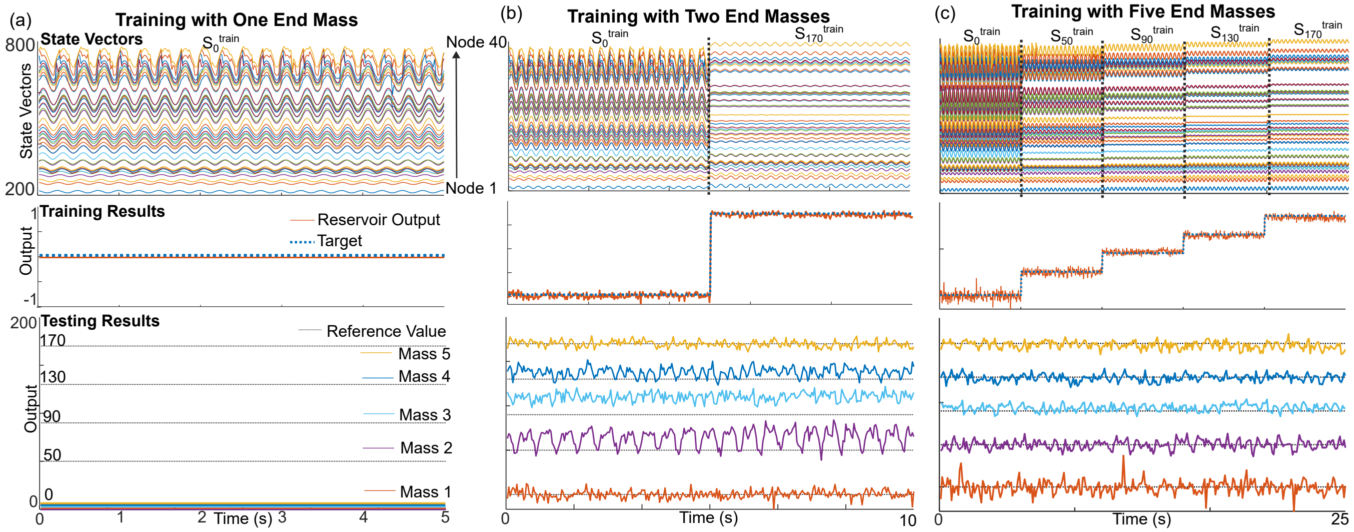


**Fig. S11.** Training the modular reservoir $C_5$ with 4 Hz harmonic base excitation using (a) one payload mass (0 g), (b) two payload masses (0 g and 170 g), and (c) all five payload masses (0 g, 50 g, 90 g, 130 g, 170 g). The first subplot shows the compiled state vectors used for training. The second subplot shows the corresponding target outputs and the reservoir training results. The third subplot shows the reservoir outputs for testing data using the trained readout weights.

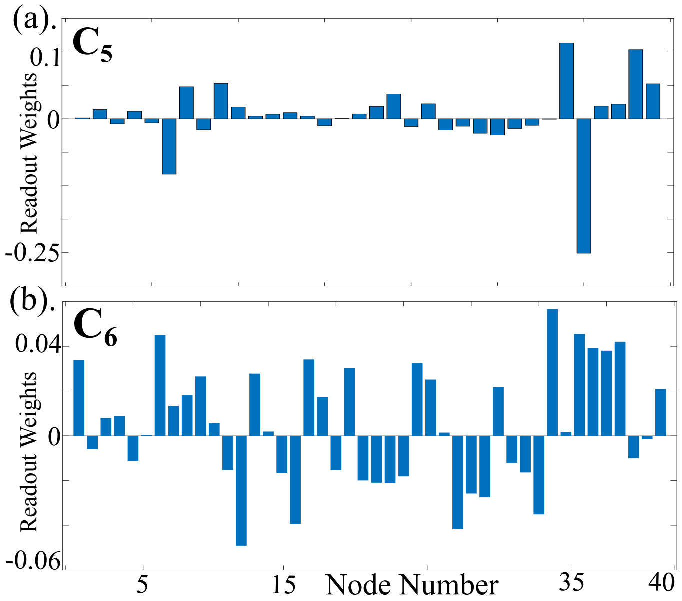


**Fig. S12.** Readout weight distribution after training for Task 2. (a) and (b) show results for Configuration 5 and 6, respectively, when they are excited by a 10 Hz input with all five payload conditions involved in training.

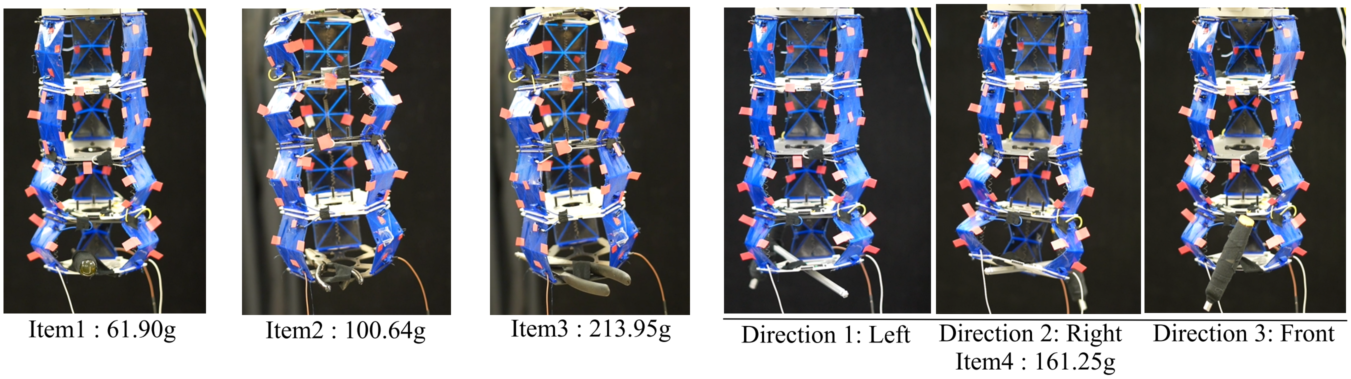


**Fig. S13.** Initial setup showing the modular arm holding four different items. Item 4 (hammer) is additionally tested in three orientations (front, left, and right).

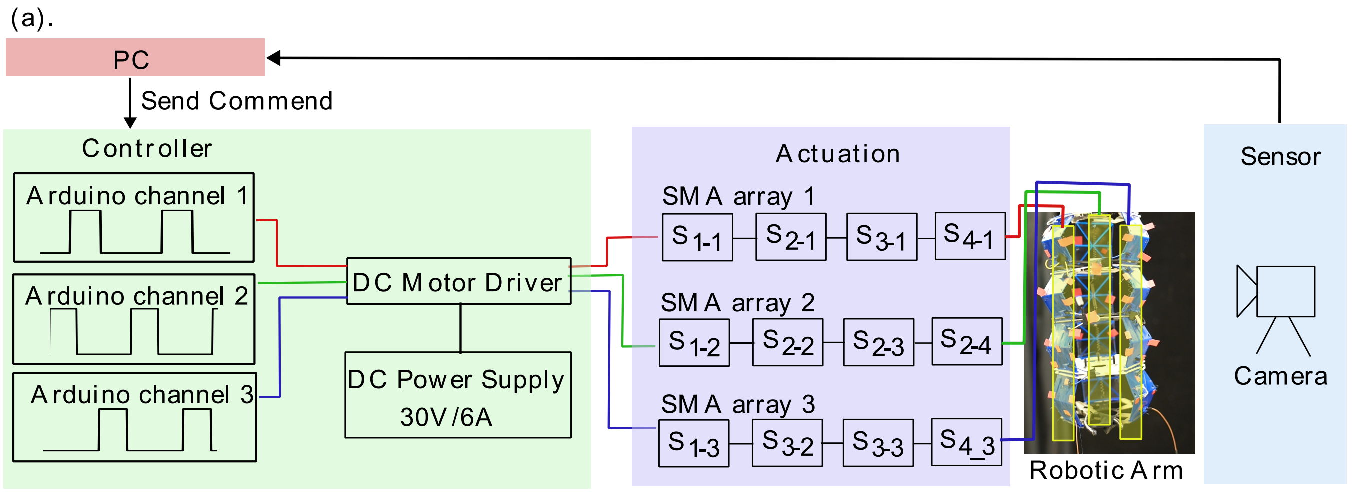


**Fig. S14.** Mechatronic design for actuating the modular arm.

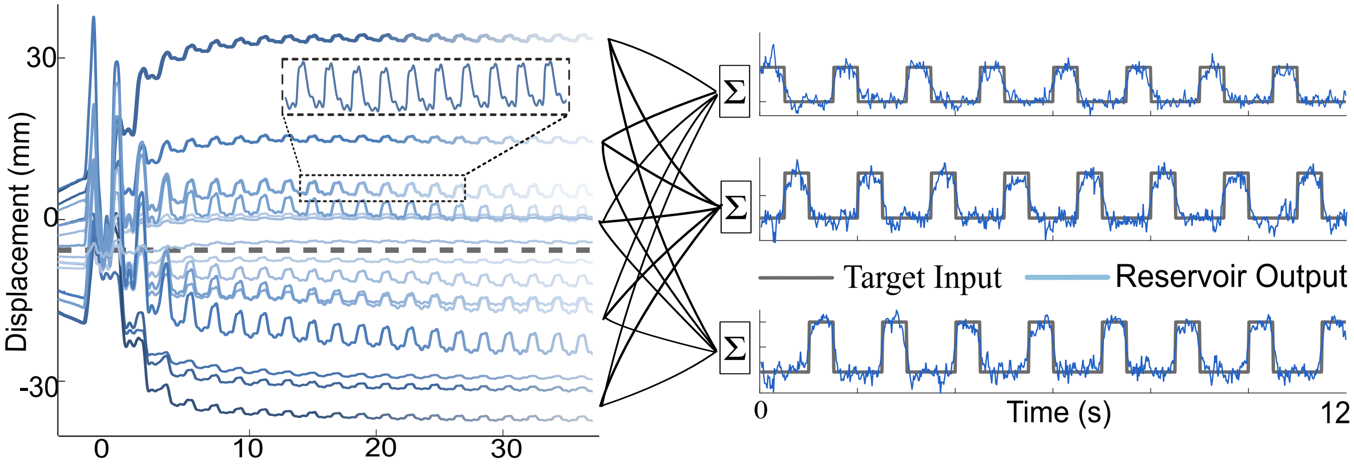


**Fig. S15.** Training process for three SMA command emulations using displacement data collected when configuration $C_7$ is actuated at frequency $f_8$ (1.5 s per cycle).

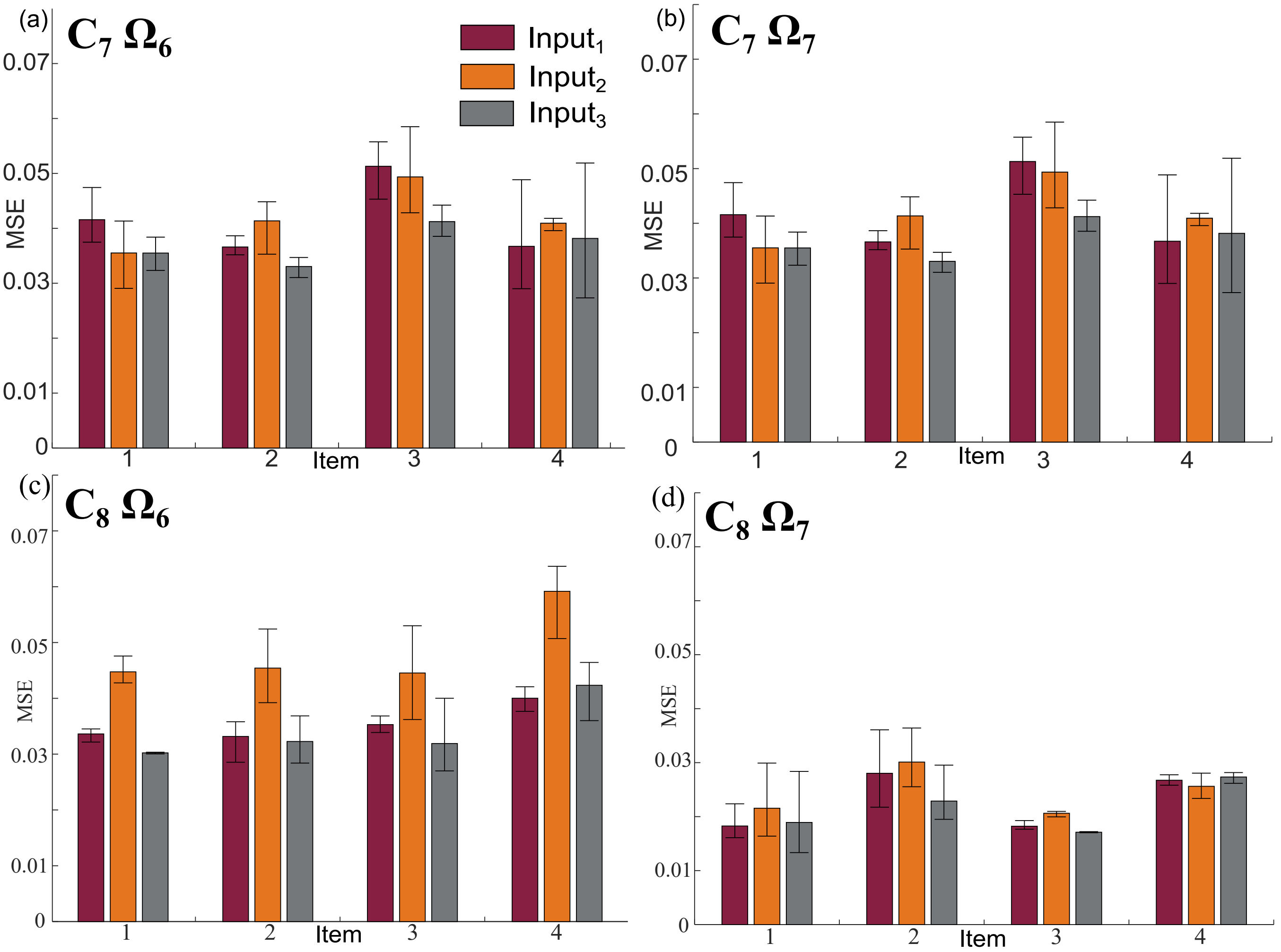


**Fig. S16.** MSE of input reconstruction tasks. (a) shows the MSE bar plot when the arm in configuration $C_7$ is actuated at frequency $f_6$ under four different payload conditions. Similarly, (b), (c), and (d) present the results for $C_7 f_7$, $C_8 f_6$, and $C_8 f_7$, respectively. Each bar represents the average MSE over 10 trials, and the error bars indicate the minimum and maximum values.

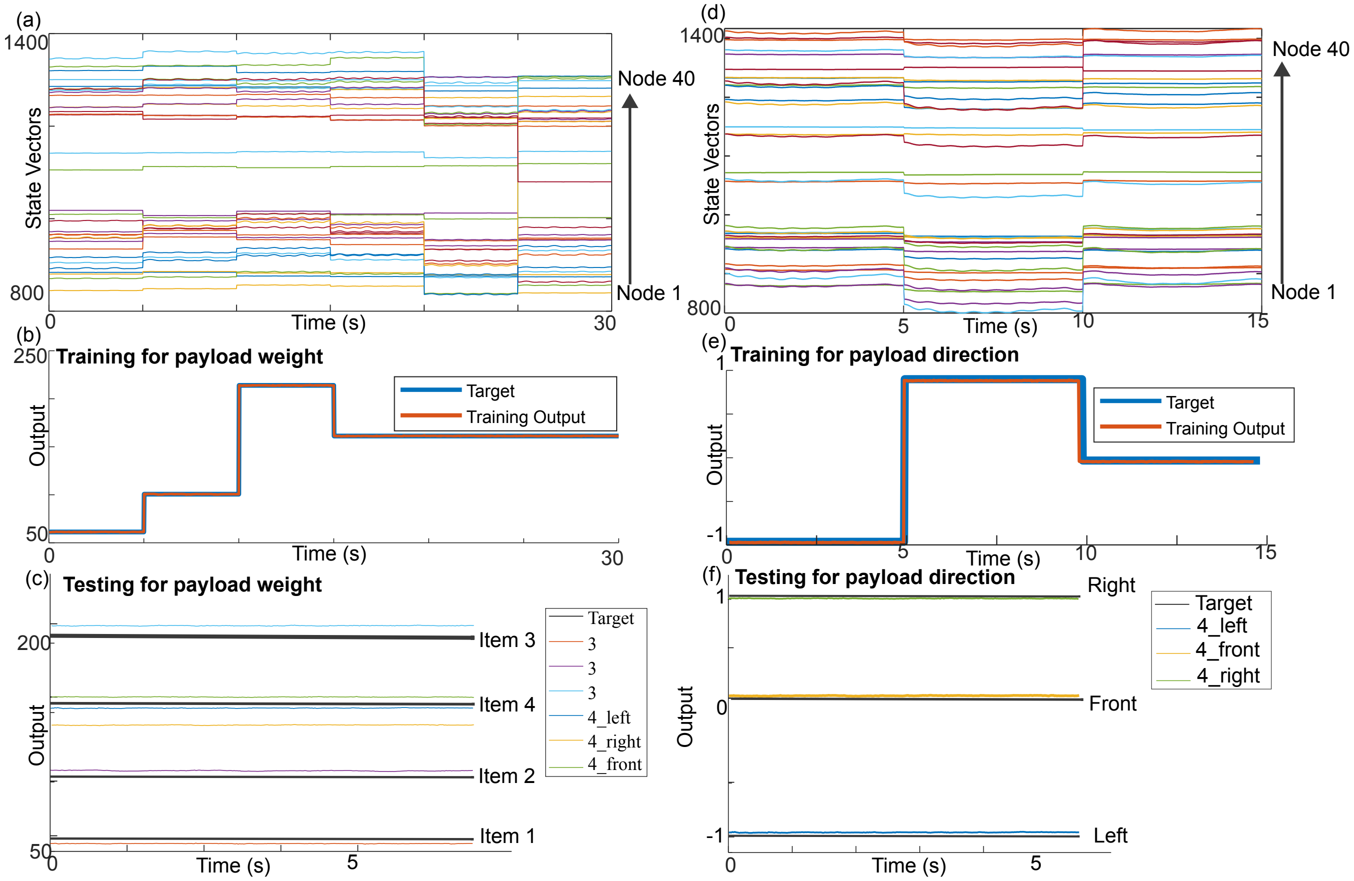


**Fig. S17.** (a-c). Training and testing results for the payload weight estimation task. (d-f). Training and testing results for the hammer orientation classification task.

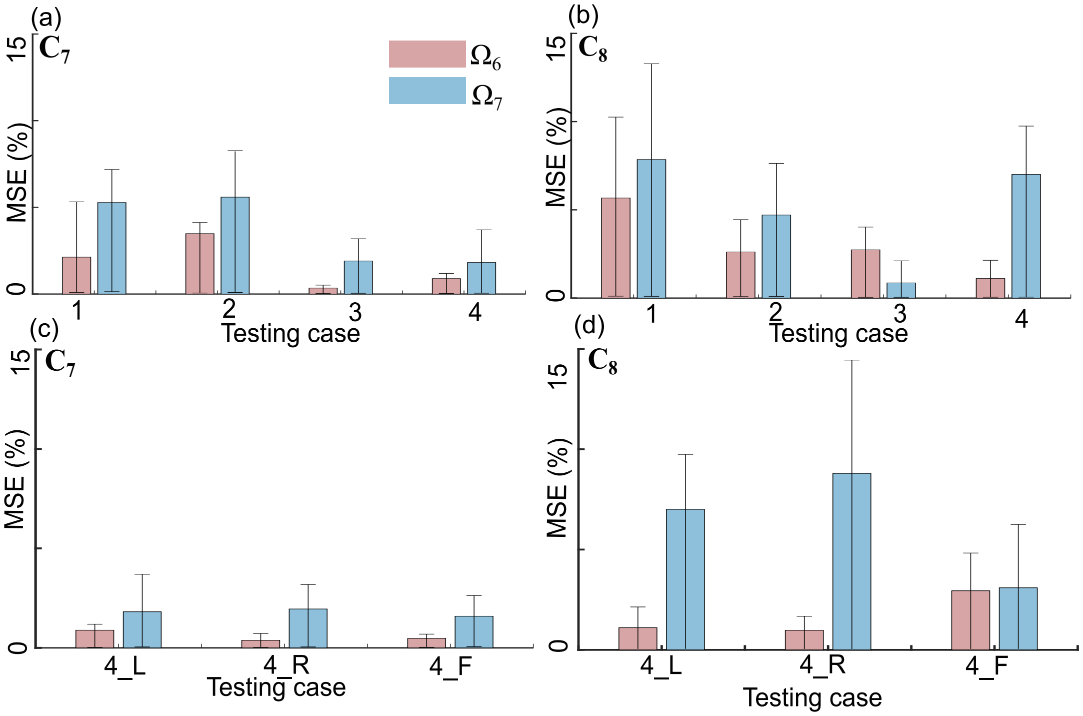


**Fig. S18.** MSE results for the payload perception tasks. (a, b) MSE of weight estimation under configurations $C_7$ and $C_8$. (c, d) MSE of orientation classification under the same configurations.

**Movie S1. NARMA Emulation with adaptive kernel**

**Movie S2. Payload estimation with adaptive kernel**

**Movie S3. Multi-tasking with modular arm**

## References


1. V Deshpande, Y Phalak, Z Zhou, I Walker, S Li, ‘golden ratio yoshimura’for meta-stable and massively reconfigurable deployment. *Philos. Transactions A* **382**, 20240009 (2024).
2. C Wringe, M Trefzer, S Stepney, Reservoir computing benchmarks: a review, a taxonomy, some best practices. *arXiv preprint arXiv:2405.06561* (2024).
3. M Inubushi, K Yoshimura, Reservoir computing beyond memory-nonlinearity trade-off. *Sci. reports* **7**, 10199 (2017).
4. K Nakajima, H Hauser, T Li, R Pfeifer, Information processing via physical soft body. *Sci. reports* **5**, 10487 (2015).
5. Z Zhou, S Li, Self-sustained and coordinated rhythmic deformations with sma for controller-free locomotion. *Adv. Intell. Syst.* **6**, 2300667 (2024).
